# Supplementary material for: Time-efficient physical activity intervention for older adolescents with disability: rationale and study protocol for the Burn 2 Learn adapted (B2La) cluster randomised controlled trial
Source: BMJ Open. 2022 Aug 10;12(8):e065321. doi: 10.1136/bmjopen-2022-065321 (PMC9379534; doi:10.1136/bmjopen-2022-065321)
Supplement: Supplementary data [file bmjopen-2022-065321supp001.pdf]

Professor David Lubans
School of Education
Faculty of Education and Arts
University of Newcastle
Callaghan NSW 2308
Phone: + 61 (0)2 4921 2049
Fax: +61 (0)2 4921 7407
Email: david.lubans@newcastle.edu.au

THE UNIVERSITY OF NEWCASTLE AUSTRALIA

Research Project: Increasing physical activity and improving fitness to support the wellbeing of senior school students

Document Version 7; dated 13/05/2020

STUDENT & PARENT INFORMATION STATEMENT

Dear student and parent/guardian,

You are invited to participate in the research project identified above which is being conducted by Prof David Lubans and colleagues from the University of Newcastle and Australian Catholic University. This research study is being funded by a National Health and Medical Research Council (NHMRC) project grant.

Why is this research being done?

Physical activity declines sharply during adolescence and findings reported in the 2016 Active Healthy Kids Australia (AHKA) Physical Activity Report Card suggest that only 18% of 12-17 year old youth meet national physical activity guidelines. The senior school years can be a time of significant stress for students, and recent national surveys show that a substantial proportion of year 11 and 12 students experience moderate to high levels of psychological distress. Regular physical activity has been shown to support young peoples' mental health, and there is growing evidence linking physical activity and fitness to cognitive functioning and academic performance. Despite these benefits, schools usually do not provide regular physical activity opportunities to students during the senior school years.

The primary aim of the study is to assess the impact of a physical activity program delivered in the school setting on students' fitness, well-being and cognitive functioning.

Who can participate in this research?

Grade 11 and 12 students from the Special Education Faculty, Callaghan College Senior Campus. We aim to recruit 20-25 students.

What choice do you have?

The program components will be delivered by teachers as part of regular school activity. This invitation relates to the evaluation of that program. Participation in the evaluation component of this study is entirely up to you and your parent(s)/guardian(s), and you can choose to participate in all, some or none of the evaluation measures. If you agree to participate you can choose to withdraw at any time. A decision not to participate or discontinuation of involvement in the study will not jeopardise your relationship with the University of Newcastle, Australian Catholic University or the school. Withdrawal from the evaluation component will not result in any disciplinary action, nor will it affect your academic grades, given that this is a purely voluntary research task.

What is involved in this study?

The program will run for 8-weeks and has been designed to improve students' physical and mental health. Program will start in Term 2 (week 6) and run into Term 3 (week 4). Consenting students will complete evaluation measures at baseline and 8-weeks. Students who do not consent to participate will not be involved in the evaluation component of the study. However, it is the school's decision to deliver the physical activity program as part of the school curriculum. Therefore, a decision not to participate in the program activities must be discussed with and decided upon by the school. The program components and evaluation measures are listed below in Table 1.

Table 1: Program components and evaluation strategies

Program components	***Evaluation of program***
**i) Interactive seminar:** Students will attend 1 interactive seminar delivered by the school champion, but supported by a member of the research team. The interactive seminar will provide an overview of Burn 2 Learn and will address relevant information regarding physical activity, mental health and cognition.<br><br>**ii) School-based exercise sessions:** Sessions will be run at school during class time. Schools will be encouraged to offer 3 sessions/week for 2 school terms (i.e., the colder months), and each session will last approximately 10-15 minutes in duration. The sessions will involve a combination of aerobic (e.g., shuttle runs, jumping jacks, boxing, dancing) and muscle strengthening exercises (e.g., push-ups, squat jumps and walking lunges), and have been designed to be fun and engaging as well as vigorous in nature.<br><br>**iii) Parental e-newsletter:** Parents will receive two e-newsletters containing information on the benefits of physical activity for academic performance and mental health and strategies to support their children's participation in physical activity during school holiday periods.<br><br>**iv) Smartphone app:** A smartphone app has been developed to enable students to complete the sessions at school and home. Android and iOS versions of the app will be available. The app includes: (i) descriptions of exercise sessions with timer, audible prompts and recording of heart rate results, (ii) ability to review session heart rate records, and (iii) self-monitoring/goal setting to promote participation in all types of physical activity.	The following measures will be taken twice (baseline and 8-weeks):<br><br>• *Health-related fitness:* Aerobic fitness will be tested using the 3 minute step test and muscular fitness will be assessed using the timed push up and sit to stand tests. Body composition will be assessed sensitively using height and weight (i.e., to calculate Body Mass Index [BMI]). Measurements will take place out of the view of other students, and students will have the choice of being assessed by either a male or female researcher.<br><br>• *Mental health:* will be measured using a questionnaire.<br><br>• *Sleep behaviour*: will be assessed using existing questionnaires.<br><br>• *Cognitive functioning:* will be assessed using computer-based tests that assess aspects of cognitive control.

How long will it take?

**Task**	**Approximate length of time**
Baseline and post-intervention questionnaires and testing	50-60 minutes at each time point
Physical activity sessions	Approximately 15 minutes/session, 3 times per week for 8-weeks.

What are the risks and benefits of participating?

The evaluation measures will be carried out by trained research assistants, and will be conducted in a sensitive manner at all times. The school-based exercise sessions will be developed by the research team and delivered by students. Based on previous studies, students will have no greater chance of injury by participating in these programs in comparison to other sports and physical activities. In the event of an injury occurring, the student will immediately be asked to stop participation, and normal school procedures for the management of injury will be followed. The student will not return to participation in the program's physical activities until clearance has been received from a suitable practitioner. If you have a health or medical condition precluding your participation in vigorous exercise, you SHOULD NOT participate in this program. If you are unsure if this applies to you, you and your parent/guardian should seek advice from a qualified medical professional (e.g., a General Practitioner) prior to consenting to participate. The program will provide students with an opportunity to increase their knowledge and skills and improve attitudes toward physical activity. Students will also benefit from participation in a variety of enjoyable exercise activities as part of the program's delivery. Questionnaires utilised during the program evaluation involve the use of a Psychological Distress Scale and other questions that individuals may find distressing. To manage this risk, students are advised on each survey that: "*Completing the questionnaires is entirely the choice of the participants. Some of the questions are of a personal nature. If you feel uncomfortable with any question you may leave it and*

move to the next question, or discontinue the survey," and are provided with the contact details for Lifeline. If your involvement in completing any of the surveys or activities in the program make you feel uncomfortable or cause you distress then please contact Lifeline on 13 11 14 and/or your School Counsellor. You can also contact the researchers (contact details at the end of this letter) to discuss your concerns confidentially.

How will the information collected be used?

The data collected from this study will be used for journal publications and conference presentations and to inform future practice for the design of valuable, evidence-based physical activity programs in schools.

How will privacy be protected?

Any personal information provided by students and parents will be confidential to the researchers. The results of the study will be published in general terms and will not allow the identification of individual students or schools. Once the data has been collected, de-identified using participant codes and entered into an electronic data file, questionnaires and other data collection sheets will be destroyed. Data will be stored for a minimum of 5 years on password protected files (only accessible to researchers).

What do you need to do to participate?

Take some time to read through this information statement and discuss your participation with a parent/guardian. All students wanting to participate in the evaluation component of this study will be required to return the accompanying consent form, signed by the student and a parent/guardian. If you consent to participate, please return the signed form as soon as possible to the school's front office, the Head Teacher of the PE faculty, or your Year Advisor. If you are 18 years or older, you will not need a parent/guardian to provide consent. However, we still encourage you to discuss your involvement with a trusted adult prior to agreeing to participate.

Further information

Following the completion of the study, the school will be sent a report describing the findings of the study. Participants can request/access a summary of the results of the research by contacting Prof. David Lubans via phone or email (contact details below). Individual results will not be given to students. If you would like further information please do not hesitate to contact Prof David Lubans by email (david.lubans@newcastle.edu.au) or phone (02 4921 2049). Thank you for considering this invitation.

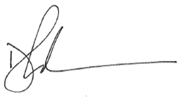

____
Prof David Lubans

Should you have concerns about your rights as a participant in this research, or you have a complaint about the manner in which the research is conducted, it may be given to the researcher, or, if an independent person is preferred, to the Human Research Ethics Officer, Research & Innovation Services, The University of Newcastle, University Drive, Callaghan NSW 2308, Australia, telephone (02) 49216333, email Human-Ethics@newcastle.edu.au.

This project has been approved by the University of Newcastle's Human Research Ethics committee, Approval number [H-2016-0424].

Professor David Lubans
School of Education
Faculty of Education and Arts
University of Newcastle
Callaghan NSW 2308
Phone: + 61 (0)2 4921 2049
Fax: +61 (0)2 4921 7407
Email: David.Lubans@newcastle.edu.au

THE UNIVERSITY OF NEWCASTLE AUSTRALIA

Research Project: Increasing physical activity and improving fitness to support the wellbeing of senior school students
STUDENT / PARENT CONSENT FORM

Chief Investigators: Prof David Lubans, Prof Philip Morgan, Prof Ronald Plotnikoff, Prof Chris Lonsdale, Prof Michael Nilsson, Dr Jordan Smith, Dr Narelle Eather

I have been given information about the project identified above and have discussed it with the student. We understand that if I consent to the student's involvement, he/she will participate in the study entitled: *Increasing physical activity and improving fitness to support the wellbeing of senior school students*.

We understand that the student will complete the following program evaluation measures: weight, height, cardio-respiratory fitness, muscular fitness, mental health, sleep behaviour, and cognitive functioning.

We have had an opportunity to ask Prof Lubans questions about the research. I have discussed this project with the student and we understand that their participation in this research is voluntary and that he/she is free to withdraw from all or part of the evaluation component at any time. His/her refusal to participate or withdrawal of consent will not affect his/her relationship with the University of Newcastle, Australian Catholic University or the school. A decision to withdraw will not result in any disciplinary action against the student, nor will it affect his/her academic grades, given that this is a purely voluntary research task.

Please discuss the project together and ensure that you are both happy for the student to participate before signing the consent form. By signing below I am indicating consent / assent to participate in this research project conducted by Prof David Lubans, as it has been described to us in the Information Statement, a copy of which I have retained.

Student name: ____

Student's signature: ________________________________ **Date:** ______________________

Student's mobile number (if applicable): ________________________
(this will be used to send a reminder regarding program session days/times).

Parent/guardian name: ____

Parent Phone: ____

Parent's Signature: _______________________________ **Date:** ______________________

Please sign the completed consent letter and return to the school office or Special Education Faculty.

Should you have concerns about your rights as a participant in this research, or you have a complaint about the manner in which the research is conducted, it may be given to the researcher, or, if an independent person is preferred, to the Human Research Ethics Officer, Research & Innovation Services, The University of Newcastle, University Drive, Callaghan NSW 2308, Australia, telephone (02) 49216333, email Human-Ethics@newcastle.edu.au.

This project has been approved by the University of Newcastle's Human Research Ethics Committee, Approval number [H-2016-0424].
