# Supplementary material for: Time-efficient physical activity intervention for older adolescents with disability: rationale and study protocol for the Burn 2 Learn adapted (B2La) cluster randomised controlled trial
Source: BMJ Open. 2022 Aug 10;12(8):e065321. doi: 10.1136/bmjopen-2022-065321 (PMC9379534; doi:10.1136/bmjopen-2022-065321)
Supplement: Author's manuscript [file bmjopen-2022-065321.draft_revisions.pdf]

BMJ Open is committed to open peer review. As part of this commitment we make the peer review history of every article we publish publicly available.

When an article is published we post the peer reviewers' comments and the authors' responses online. We also post the versions of the paper that were used during peer review. These are the versions that the peer review comments apply to.

The versions of the paper that follow are the versions that were submitted during the peer review process. They are not the versions of record or the final published versions. They should not be cited or distributed as the published version of this manuscript.

BMJ Open is an open access journal and the full, final, typeset and author-corrected version of record of the manuscript is available on our site with no access controls, subscription charges or pay-per-view fees (http://bmjopen.bmj.com).

If you have any questions on BMJ Open's open peer review process please email info.bmjopen@bmj.com

BMJ Open

Time-efficient physical activity intervention for older adolescents with disability: Rationale and study protocol for the Burn 2 Learn adapted (B2La) cluster randomised controlled trial

Journal:	*BMJ Open*
Manuscript ID	bmjopen-2022-065321
Article Type:	Protocol
Date Submitted by the Author:	06-Jun-2022

Complete List of Authors:	Kable, Toby; The University of Newcastle, School of Education<br>Leahy, Angus; The University of Newcastle, PRC for Physical Activity and Nutrition<br>Smith, Jordan ; The University of Newcastle, School of Education<br>Eather, Narelle; The University of Newcastle, School of Education<br>Shields, Nora; La Trobe University,<br>Noetel, Michael; Australian Catholic University - Blacktown Campus, Institute for Positive Psychology and Education; Australian Catholic University - Blacktown Campus, School of Behavioural and Health Sciences<br>Lonsdale, Chris; Australian Catholic University - Blacktown Campus<br>Hillman, Charles; Northeastern University, Department of Psychology; Northeastern University, Department of Physical Therapy<br>Reeves, Penny; University of Newcastle Hunter Medical Research Institute, Health Research Economics; The University of Newcastle, School of Medicine and Public Health<br>Oldmeadow, Christopher; The University of Newcastle Hunter Medical Research Institute<br>Kennedy, Sarah; Western Sydney University, School of Health Sciences<br>Boyer, James; NSW Department of Education, School Sport Unit<br>Stimpson, Leisl; Special Olympics Australia, Education & Training<br>Comis, Pierre; Special Olympics Australia<br>Roche, Laura; The University of Newcastle, School of Education<br>Lubans, David; The University of Newcastle, School of Education

Keywords:	PUBLIC HEALTH, SPORTS MEDICINE, STATISTICS & RESEARCH METHODS

SCHOLARONE™
Manuscripts

Time-efficient physical activity intervention for older adolescents with disability: Rationale and study protocol for the Burn 2 Learn adapted (B2La) cluster randomised controlled trial

Toby Kable[1], Angus A. Leahy[1], Jordan J. Smith[1], Narelle Eather[1], Nora Shields[2], Michael Noetel[3], Chris Lonsdale[4], Charles H. Hillman[5,6], Penny Reeves[7,8], Christopher Oldmeadow[7,8], Sarah G. Kennedy[9], James Boyer[10], Leisl Stimpson[11], Pierre Comis[11], Laura Roche[12], David R. Lubans§[1]

[1]Centre for Active Living and Learning, College of Human and Social Futures, University of Newcastle, Callaghan, New South Wales, Australia

[2]Department of Physiotherapy, Podiatry and Prosthetics and Orthotics, La Trobe University, Melbourne, Victoria, Australia

[3]School of Behavioural and Health Sciences, Faculty of Health Sciences, Australian Catholic University, Banyo, New South Wales, Australia

[4]Institute for Positive Psychology and Education, Faculty of Health Sciences, Australian Catholic University, North Sydney, New South Wales, Australia

[5]Department of Psychology, Northeastern University, Boston, Massachusetts, United States of America

[6]Department of Physical Therapy, Movement and Rehabilitation Sciences, Northeastern University, Boston, Massachusetts, United States of America

[7]School of Medicine and Public Health, College of Health, Medicine and Wellbeing, University of Newcastle, Callaghan, New South Wales, Australia

[8]Hunter Medical Research Institute, New Lambton Heights, NSW, Australia

[9]School of Health Sciences, Translational Health Research Institute, Western Sydney University, Darug Country, New South Wales, Australia

[10]New South Wales Department of Education, Turrella, New South Wales, Australia

[11]Special Olympics Australia, Concord West, New South Wales, Australia

[12]Special and Inclusive Education, School of Education, College of Human and Social Futures, University of Newcastle, Callaghan, New South Wales, Australia

For peer review only - http://bmjopen.bmj.com/site/about/guidelines.xhtml

§Corresponding author details
Professor David Lubans
Centre for Active Living and Learning
School of Education, University of Newcastle
Callaghan, NSW, Australia 2308
P: +612 4921 2049
E: David.Lubans@newcastle.edu.au

Key words: Exercise; High-intensity interval training; Adolescents; Disability; Special Education; Behaviour change; Resistance Training; Mental health; Cognition
Word count: 4,900 (1 table, and 2 figures)

For peer review only

For peer review only - http://bmjopen.bmj.com/site/about/guidelines.xhtml

ABSTRACT

Introduction: Physical activity declines during adolescence, with the lowest levels of activity observed among those with disability. Schools are ideal settings to address this issue; however, there have been few school-based interventions specifically designed and evaluated for older adolescents with disability. Our aim is to investigate the effects of a school-based physical activity program, involving high-intensity interval training (HIIT), on the physical, mental, and cognitive health of older adolescents with disability.

Methods and Analysis: The Burn 2 Learn *adapted* (B2La) intervention will be evaluated using a two-arm, parallel group, cluster randomised controlled trial with allocation occurring at the school level (treatment or wait-list control). Secondary schools will be recruited in two cohorts from New South Wales, Australia. The trial will aim to recruit 300 older adolescents (aged 15-19 years) with disability from 30 secondary schools (10 in Cohort 1 and 20 in Cohort 2). Schools allocated to the intervention group will deliver two HIIT sessions per week during scheduled specialist support classes. The sessions will include foundational aerobic and muscle strengthening exercises tailored to meet student needs. Teachers will be provided with training, resources, and support to facilitate the delivery of the B2La program. Study outcomes will be assessed at baseline, 6-months (primary endpoint) and 9-months. The primary outcome is functional capacity assessed using the 6-Minute Walk/Push Test. Secondary outcomes include physical activity, muscular fitness, body composition, cognitive function, quality of life, physical literacy, and on-task behaviour in the classroom. Detailed economic and process evaluation will also be conducted to determine program efficiency, acceptability, implementation, adaptability, and practicality.

Ethics and dissemination: This study has received approval from the University of Newcastle (H-2021-0262) and the New South Wales (NSW) Department of Education (SERAP: 2021257) human research ethics committees.

Trial registration: Australian New Zealand Clinical Trials Registry Number: ACTRN12621000884808.

For peer review only

For peer review only - http://bmjopen.bmj.com/site/about/guidelines.xhtml

Strengths and limitations of this study

Strengths of this study include:

- The cluster randomised controlled trial design adequately powered to detect meaningful changes in the primary outcome.
- Informed by our pilot work, we have tailored the intervention and assessment processes to increase accessibility for the unique study population.
- The Burn 2 Learn adapted intervention has been designed in consultation with adolescents with disability, and key stake holders (i.e., NSW Department of Education and Special Olympics Australia).

Limitations of the study include:

- Having a unique study population where due to physical and intellectual limitations, not all participants will be able to complete all measures.
- It might not be possible to blind assessors for all outcomes, as group allocation is often revealed by research participants and teachers during post-test assessments in school-based trials.

For peer review only

For peer review only - http://bmjopen.bmj.com/site/about/guidelines.xhtml

INTRODUCTION

Disability is an umbrella term used to describe impairments (i.e., problems in body function or structure), activity limitations (i.e., difficulty in performing activities), and participation restrictions (i.e., difficulty engaging in life situations) [1]. Disability is recognised as a worldwide public health and human rights issue with 15% of the global population estimated to be living with disability [2]. These individuals often face widespread barriers to accessing health and related services, and have poorer health outcomes than those without disability [3]. As noted in the WHO's Global Disability Action Plan, the burden of disability can be reduced by addressing the determinants of health, including participation in physical activity [3]. Individuals with disability are typically less physically active [4] and more likely to have co-occurring chronic and complex lifestyle diseases [5, 6] than those without disability.

Young people with disability face many common and unique barriers to participation in physical activity. Previous research has identified personal (i.e., injury, lack of skills and time to exercise), social (i.e., unsupportive peers and parents), and environmental (i.e., inadequate accessibility and lack of appropriate programs) barriers to participation in this population [7, 8]. Conversely, factors shown to facilitate participation include having the time available, being involved in programs that are adaptable, and exercising in a group with people of a similar age [9]. Parents also play a critical role in determining whether young people with disability are physically active [8]. While parents typically acknowledge the value of physical activity and want their children to be active, many also express concerns about time commitments, balancing the needs of family members, and the suitability of programs [8]. To date, the majority of physical activity interventions targeting young people with disability have been conducted in clinical, community, and home settings [10].

Schools are ideal for physical activity promotion, as they provide access to the adolescent population and have the necessary equipment, facilities, and personnel to deliver programs [11]. Physical education (PE) is the primary means of physical activity promotion in schools, and there is a large body of research focusing on the inclusion of children and adolescents with disability in PE classes [12]. While teachers typically advocate for inclusion in PE, many lack the confidence and competence to successfully involve students with disability in ways that truly benefit their physical literacy [12]. Moreover, students with disability often feel marginalised when participating in mainstream PE classes, commonly reporting feelings of social isolation, bullying, and negative social comparisons [13]. Regardless, simply

For peer review only - http://bmjopen.bmj.com/site/about/guidelines.xhtml

integrating students with disability into mainstream PE is not enough to produce meaningful changes in health, particularly in the final years of school where there is no mandatory physical activity for students [14]. In light of this, there is a need for innovative school-based physical activity interventions designed specifically for older adolescents with disability.

Implementing health promotion interventions with older adolescents is challenging, and lack of time is a major barrier to physical activity promotion for this age group [15]. For older adolescents with disability, the final years of school also involves participation in programs facilitating transition into post school pathways (e.g., community access and transition to work programs). Given that adolescents with disability have many competing needs as they prepare for life after schooling, school-based physical activity programs will have the best chances of adoption if they do not require a substantial time commitment. However, physical activity programs that provide only a small 'dose' of activity are also unlikely to result in meaningful benefits for those participating; that is, unless the physical activity offered is of sufficient high intensity.

High-intensity interval training (HIIT) is a time-efficient strategy for improving physical, psychological, and cognitive health in typically developing children and adolescents [16, 17]. HIIT sessions generally consist of several short bouts of vigorous activity interspersed with brief periods of light activity or rest. HIIT allows participants to experience similar effects to other modes of exercise, in less time. Previous studies evaluating school-based HIIT programs for adolescents with disability have shown improvements in physical health (i.e., body composition, aerobic fitness), but have been delivered by physiotherapists and experienced physical educators [18, 19]. Such programs have limited scalability due to ongoing costs required for intervention implementation. To enhance program scalability, having classroom teachers implement the intervention has greater potential to change school practice.

We recently conducted a large-scale evaluation of the first teacher-facilitated school-based HIIT intervention, known as Burn 2 Learn (B2L), for older adolescents in mainstream schools [20-23]. Briefly, teachers were trained to deliver 2-3 HIIT sessions per week for 16 weeks during students' regular academic lessons. Positive effects were observed for the primary outcome of cardio-respiratory fitness (CRF), as well as a range of secondary outcomes (e.g., muscular fitness, mental health, and classroom engagement). Following the

For peer review only - http://bmjopen.bmj.com/site/about/guidelines.xhtml

success of the intervention, our research team was approached by a local school to adapt the B2L intervention for students with disability. We subsequently conducted a pilot study of the Burn 2 Learn adapted (B2La) program in one secondary school (NSW) [24]. We found it was feasible to train special and inclusive education teachers to deliver B2La sessions, which were well received by teachers and students. We also found preliminary support for program efficacy for improving functional capacity and muscular fitness. Following our successful feasibility study, we partnered with the NSW Department of Education and Special Olympics Australia to refine and evaluate B2La in a larger effectiveness trial.

Study objectives

The primary aim of this trial is to determine the effect of the B2La intervention on the functional capacity (primary outcome) of older adolescents with disability. Secondary outcomes include physical activity, muscular fitness, body composition, cognitive function, mental health, physical literacy, and on-task classroom behaviour. We will also conduct a detailed economic and process evaluation to determine cost effectiveness, program efficiency, acceptability, implementation, adaptability, and practicality.

METHODS

Study design

The trial is registered with the Australian New Zealand Clinical Trials Registry (ACTRN12621000884808). The design, conduct and reporting of this trial will adhere to the CONSORT [25] guidelines. The B2La intervention will be evaluated using a two-arm parallel group cluster randomised controlled trial (RCT) with a treatment group and wait-list control group. Assessments will occur at baseline, 6- (primary endpoint) and 9-months from baseline (secondary endpoint). The RCT will be conducted with two cohorts, one starting in 2022 (10 schools; 5 intervention and 5 control), and another starting in 2023 (20 schools; 10 intervention and 10 control). Baseline data collection will occur in the school term preceding intervention delivery (i.e., Term 1 [February to April 2022 and 2023]. The intervention will be delivered during Terms 2, 3 and 4 [April to November 2022 and 2023]. Immediate post-intervention data collection (i.e., ~6-months) will occur at the end of Term 3 (August to September 2022 and 2023), and follow-up assessments (i.e., ~9-months) will be completed in Term 4 (November to December 2022 and 2023).

For peer review only - http://bmjopen.bmj.com/site/about/guidelines.xhtml

School recruitment and selection

NSW Government, Catholic, and Independent secondary schools with student cohorts that include older adolescents (i.e., Grades 10 to 12, students aged 15-19) with disability are eligible to participate. Schools will include both mainstream schools with specialist support classes and Schools for Specific Purposes (SSPs). SSPs are dedicated schools for students with moderate-to-high learning and support needs. Eligible schools will be identified, and an expression of interest directed to the school principal. Interested schools will then liaise with the project manager to address any questions or concerns they have prior to returning informed consent.

Participants

Students at the study schools are eligible to participate if they are: (i) in Grades 10 to 12 (15-19 years) and identify as living with disability (including neurodevelopmental disability, physical, intellectual, or sensory disabilities), (ii) able to follow simple verbal instructions in English (as determined by the Index of Social Competence) [26], and (iii) able to participate in vigorous intensity exercise (wheelchair users will be eligible). We will aim to recruit 10 students from each school. We will also recruit two special and inclusive education teachers per school, who will act as school champions and facilitate the delivery of B2La sessions. Special and inclusive education teachers develop and deliver specialised learning programs for students with a range of disabilities and learning difficulties.

Sample size and power calculation

Power calculations were based on the primary outcome of functional capacity, assessed using the 6-Minute Walk Test (6MWT), which has good reliability in adolescents with disability (ICC =0.82) [27]. A 6-Minute Push Test will be administered for wheelchair users. Although adolescent data are lacking, studies conducted among adult populations with chronic health conditions have reported minimal clinically important differences (MCID) of 24 to 44 metres using the 6MWT [28]. In our pilot study, we observed a large increase in distance covered from baseline to immediately after the intervention period (163 ± 130.9 m). However, our pilot study did not include a control group and effects are typically smaller in effectiveness trials compared with pilot studies [29]. Based on the pilot data, we estimate a treatment effect of 80m will represent a MCID in our population. Through simulations (n=10,000) and using data from our pilot study (i.e., baseline post-test correlation of r = 0.60, standard deviation of 90m and intraclass correlation of 0.2), we have determined we will require a sample of 30

For peer review only - http://bmjopen.bmj.com/site/about/guidelines.xhtml

schools with 7 participants per school. This sample size will be enough to detect a MCID of 80m with 90% power at a 5% significance level. Allowing for 30% loss to follow-up at 6-months we will recruit 10 students from 30 schools (total sample size of 300).

Blinding and randomisation

Randomisation will occur within each cohort once consenting schools have completed baseline assessments. Schools will be matched as closely as possible based on the following characteristics in this order: (i) school type (i.e., mainstream school support class/SSP), (ii) school sector (i.e., Government/Catholic/Independent), (iii) geographic location (i.e., region, rural/urban, coastal/inland), and (iv) student population educational advantage (i.e., using the Index of Community Socio-Educational Advantage [ICSEA]) [30]. Matched schools will be randomised to the intervention or wait-list control group using a random number producing algorithm by an independent statistical analysis service – Clinical Research Design, Information Technology and Statistical Support (CREDITSS) run by the Hunter Medical Research Institute. One school from each pair will be allocated to the B2La condition and the other to the wait-list control condition. Schools randomised to the intervention group will deliver the B2La program during the study period, whereas schools allocated to the wait-list control group will continue with usual school practice (i.e., normal curricular lessons) for the duration of the study period and will receive the intervention the following year. We decided to use a wait-list control design, rather than an attention-matched placebo because: (i) our research team will have minimal contact with students, and (ii) our findings will have greater external validity, as participants in the control group will receive 'usual practice'.

Patient and public involvement

Following the B2L cluster RCT, our research team was asked to adapt the intervention for students with disability. We subsequently conducted a feasibility study in one secondary schools in Newcastle (N = 16 students) [24]. Participating students and teachers were invited to provide feedback on the intervention and suggestions for further improvement. This feedback was then used to refine intervention components (e.g., exercise sessions) and develop implementation strategies (e.g., professional learning for teachers). We then partnered with the NSW Department of Education and Special Olympics Australia to create B2La. We conducted further testing with teachers and students with disability before progressing to this trial.

For peer review only - http://bmjopen.bmj.com/site/about/guidelines.xhtml

For peer review only

Intervention

Intervention delivery

The B2La intervention will be delivered in four phases:

1. Laying the foundation (weeks 1 to 4)
2. Developing a routine (weeks 5 to 9),
3. Maintaining student interest (weeks 10 to 16), and
4. Moving towards independence (week 17 onwards).

In Phase 1-3 (16 weeks), teachers will facilitate the delivery of at least two HIIT sessions/week during lesson-time. Phase 1 will start with a 4-week block to familiarise students with the B2La session structure and support resources, and to develop the foundational exercise skills that are used within the HIIT sessions utilising B2La technique cards (Figure 1). During this phase, students will participate in two HIIT workouts including: Indoor HIIT and Power HIIT (as shown in Figure 2). These basic HIIT workouts do not require additional sport equipment or partner interaction which will assist teachers and students to familiarise themselves with the sessions.

In Phases 2 and 3 (weeks 5 to 16) the number of foundational exercises used within the HIIT sessions will increase as students become more confident with the exercises and session routine. There will be an increase in vigorous activity duration within the HIIT sessions and the opportunity to introduce more novel themed HIIT workouts including: Soccer HIIT and Basketball HIIT (Phase 2), and Judo HIIT, Cricket HIIT and Custom HIIT (Phase 3). During Phases 2 and 3, teachers will be encouraged to increase the amount of autonomy provided to students (e.g., choice of B2La session), as appropriate based on their classes' interest and progress throughout Phase 1. In Phase 4 (week 17 onwards), students will be encouraged to engage in physical activity sessions of interest outside of school (e.g., at home, the park), but teachers may continue to facilitate B2La sessions during lessons if they choose. Students will have their own B2La goal setting activity booklet.

Intervention components

B2La is a multi-component intervention that includes the following components: student information seminar, school-based HIIT sessions, smartphone application (app), goal setting activity booklet, and parental support videos.

For peer review only - http://bmjopen.bmj.com/site/about/guidelines.xhtml

Student information seminar: Delivered by teachers, the seminar will provide an overview of B2La and focus on the barriers and benefits of physical activity for adolescents with disability [4] as well as evidence-based behaviour change techniques (e.g., self-monitoring and goal setting). Teachers will be provided with a PowerPoint presentation template with embedded videos developed specifically for this project.

School-based exercise sessions: Sessions will be delivered during scheduled 'Learning Support Lessons'; a time-period when adolescents with disability are working separately to those without disability (for those attending mainstream schools). Students with special needs in Australian mainstream secondary schools typically attend three, 2-hour support lessons per week. Teachers will be asked to facilitate the delivery of at least two exercise sessions per week in Phases 1-3. Adapted from the original B2L intervention, teachers/participants will be able to select from pre-designed themed HIIT workouts that include a combination of foundational resistance exercises (i.e., push up, squat, front support, lunge) and aerobic exercises (i.e., shuttle run, high knees running on the spot). The sessions last 10 to 20 minutes including an appropriate warm-up. Due to the wide range of abilities expected from the participants, teachers will be encouraged to tailor the specific types of exercises and task complexity for each student. The task complexity and variations within the HIIT sessions will progressively increase over the study period. To monitor exercise intensity, students will be equipped with heart rate sensors (Polar Verity Sense) that will pair with a purpose-built iPad application using Bluetooth connectivity. Students will be encouraged to reach a target intensity of ≥80% of age-predicted heart rate maximum during the work intervals. As demonstrated in our feasibility study, this heart rate target is achievable for students with disability [24].

For peer review only - http://bmjopen.bmj.com/site/about/guidelines.xhtml

Smartphone application: Teachers and students will be provided with access to a bespoke smartphone application (hereafter 'app') available via both Android and iOS operating systems. The app includes: (i) a teacher version that allows whole-class heart rate monitoring during 'class' sessions, (ii) descriptions and depictions of exercise sessions, (iii) options for 'solo' or 'group' sessions, (iv) timer, audible prompts and display of heart rate using Bluetooth-synced heart rate monitors during HIIT sessions, and (v) personalised reports outlining heart rate. For students who do not own a smartphone, access to the B2L app will be provided to parents. During the professional development workshop, teachers will be encouraged to deliver school-based sessions using the teacher version that allows whole-class heart rate monitoring. Students will be encouraged to use the app during activity sessions outside of the school setting.

Parent support videos: Parents will receive two e-newsletters containing links to video overviews of B2La, the benefits and barriers of physical activity for individuals with disability, and strategies to support their children's participation in physical activity outside of school. The e-newsletters will be delivered to parents in Phase 1 and Phase 2 of the intervention.

Intervention conceptual model and theoretical frameworks

B2La was guided by the conceptual model proposed by Lubans and colleagues [31], which includes four complementary tenets that are fundamental to the successful scale-up of adolescent HIIT interventions.

Opportunity: The B2La sessions will be delivered during scheduled 'Specialist Support Classes' (students typically attend 3 x 2-hour support lessons/week). These classes cater for students with moderate-to-high learning and support needs, including students with intellectual disability, mental health issues, autism, physical disability, sensory impairment, and behaviour disorders. Based on our formative research with teachers and the NSW Department of Education, 'Specialist Support Classes' represent an ideal opportunity [32] for the delivery of B2La.

Design: The school-based exercise sessions will provide participants with opportunities to collaboratively develop their exercise competence and confidence. Participants will also be provided with opportunities to design and run their own HIIT sessions. The study information

For peer review only - http://bmjopen.bmj.com/site/about/guidelines.xhtml

seminar will focus on the benefits and barriers of physical activity for individuals with disability [4] as well as evidence-based behaviour change techniques.

Delivery: B2La has been guided by Self-Determination Theory (SDT) to enhance students' autonomous motivation for physical activity [33]. Aligned with SDT, teachers will be provided with training and support to deliver the B2La sessions using the 'Supportive, Active, Autonomous, Fair and Enjoyable' (SAAFE) principles [34]. Participants' need for autonomy will be satisfied by providing opportunities for choice within sessions (e.g., type of activity, music playing, and training partner). Competence will be satisfied using positive and skill-specific feedback from teachers and a focus on effort over performance (via heart rate feedback), and through the provision of resources designed to support the accessibility, engagement, and development of exercise skills (e.g., technique cards, B2L app). Teachers will utilise a variety of strategies to enhance group cohesion and satisfy students' needs for relatedness during HIIT sessions (i.e., encouraging supportive behaviour among students such as 'high fives' and facilitating partner work) [35].

Support: The implementation of B2La will be supported by the Consolidated Framework for Implementation Research (CFIR) [36]. Strategies used to facilitate the implementation of the B2La intervention will cover the five CFIR domains: intervention characteristics, outer setting (educational authorities), inner setting (schools), characteristics of individuals (teachers), and the implementation process (see Table 1).

Table 1: Strategies used to facilitate implementation in the Burn 2 Learn adapted intervention

**Domains**	**Constructs**	**Strategies**
**B2La intervention characteristics**	Evidence strength and quality	Findings from *B2L* cluster RCT and *B2La* feasibility study used in promotional and training materials.
	Adaptability	Flexible intervention delivery model (i.e., during class-time, breaks, before or after school) requiring minimal access to facilities (i.e., can be done in the classroom) and equipment (i.e., body weight exercises).
	Complexity	Time-efficient intervention requiring only two or three 15-minute sessions per week.
	Design quality and packaging	*B2La* program resources developed by professional graphic designer. Multimedia designed using evidence-based principles for learning.
**Outer setting (Educational authorities)**	Partnerships and investment	Partnership with the NSW Department of Education and Special Olympics Australia.
	External policy and incentives	Professional learning accreditation with state-based educational standards authority.

For peer review only - http://bmjopen.bmj.com/site/about/guidelines.xhtml

**Inner setting (Schools)**	School culture	Teachers will be encouraged to give a presentation to school staff focused on the benefits of activity for mental health and academic outcomes.
	Leadership engagement	Teachers and external change agents will meet with the school principal to ensure commitment.
	Equipment	Schools provided with a basic equipment pack (~$2,000 AUD).
	Relative priority	Promoted to schools as strategy to improve cognitive function and mental health.
**Characteristics of individuals (Teachers)**	Self-efficacy, knowledge, and beliefs (teacher)	Full day professional development workshop provided for teachers. Online version of workshop available.
	Perceived barriers (students)	Designed to be time efficient, and motivating for students, through the SAAFE teaching principles.
**Implementation in process**	Planning for implementation	Teachers required to complete an action plan to support B2La implementation in their school.
	Champions	Recruitment of two school champions at each intervention school.
	External support agents	Schools will be allocated external change agent, who will visit twice for planning and evaluation.
	Evaluation and feedback	External change agents will conduct session observations and provide feedback to teachers.

Note. B2La = Burn 2 Learn *adapted*

Teachers recruited as school champions will attend a one-day professional learning workshop led by members of the research team. The workshop will provide teachers with the training and resources needed to facilitate school-based HIIT sessions. The workshop will involve a combination of theoretical (i.e., program rationale, benefits of HIIT, school implementation plan) and practical (e.g., participation in a B2La HIIT session, peer assessment of exercise technique, and overview of how to use program resources) activities.

Measures and data collection

Trained research assistants, blinded to group allocation at all time-points, will conduct assessments for the primary outcome. Questionnaires will be completed with the assistance of research assistants using electronic tablets. Physical assessments will be conducted in a sensitive manner by a research assistant of the same sex where possible. Standard demographic information (e.g., age, sex, ethnicity, country of birth, residential postcode, and parent/caregivers' education level) will be collected at baseline. All measurements will be conducted at baseline, 6-months post-baseline (primary endpoint) and 9-months post-baseline. The only exception will be students' on-task behaviour which will be assessed at baseline and mid-intervention (3-months post-baseline), and cognitive function which will be assessed at baseline and 6-months only. Of note, due to physical and intellectual limitations,

For peer review only

For peer review only - http://bmjopen.bmj.com/site/about/guidelines.xhtml

not all participants will be able to complete all measures and modifications will be made as necessary.

Primary outcome

Functional capacity. Consistent with previous physical activity interventions targeting youth with disability [37], our primary outcome is functional capacity, assessed using the 6MWT [38], which has good reliability in adolescents with disability (ICC =0.82) [27]. The 6-Minute Push Test will be used for students who self-propel a wheelchair [39]. Students will be instructed to cover as much distance as possible in 6 minutes and the distance (in meters) covered will be documented.

Secondary outcomes

Physical activity. Participants will be instructed to wear an ActiGraph GT9X Link accelerometer on their non-dominant wrist for 24 hours/day (even when bathing, swimming, and sleeping) for a period of seven consecutive days (3-day minimum wear time). School hour, weekday and weekend (i.e., mean minutes per day) physical activity will be calculated separately, using existing thresholds for categorising physical activity intensity [22].

Muscular fitness. Lower body muscular endurance will be assessed using the 30 second sit-to-stand test [40]. From a seated position, students will attempt to stand up and sit back down on a 44cm high bench seat as many times as possible in 30 seconds [18]. A modified version of the 90-degree push-up test will be used to assess upper body muscular endurance [41]. All students will be instructed to perform as many push-ups as possible on their knees.

Body composition. Body weight and height will be measured using a portable digital scale (A&D Medical UC-352-BLE Digital Scales) and a portable stadiometer (Seca 213 Portable Height Measuring Rod Stadiometer), respectively. Body mass index (BMI) will be calculated using the standard formula (weight[kg]/height[m]^2). Age- and sex-specific BMI z-scores will be calculated, and participants will be classified into weight categories according to International Obesity Task Force cut-offs [42].

For peer review only - http://bmjopen.bmj.com/site/about/guidelines.xhtml

Cognitive function. This will be assessed with electronic tablets using the cognitive portion of the National Institutes of Health (NIH) Toolbox [43]. The Toolbox has been used with children and adults with Fragile X syndrome, Down syndrome, and intellectual disabilities, with tests demonstrating good to excellent reliability and feasibility [43, 44]. Participants will complete the Flanker (inhibition), list sorting (working memory) and dimensional change card sort (cognitive flexibility) tasks.

Quality of life. Health-related quality of life will be assessed using the Child Health Utility 9-Dimensions [45], which includes 9-items (worried, sad, pain, tired, annoyed, schoolwork or work, sleep, daily routine, and activities) each item scored on a 5-point scale.

Physical literacy. Autonomous motivation for physical activity will be assessed using identified and intrinsic subscales from the 'Behavioural Regulations in Exercise Questionnaire-2 [46]. Confidence will be assessed using the validated 6-item High-Intensity Interval Training Self-efficacy Questionnaire [47]. Competence will be assessed using video analysis of a selection of skills from the Resistance Training Skills Battery (i.e., push-ups, lunge, squat, and front support chest touch), which has been validated among typically developing adolescents [48] and among children with varying degrees of motor skill proficiency [49].

Externalising behaviours. Teachers will complete a Student Behaviour Questionnaire [50] for each student at baseline, 6- and 9-months. The questionnaire consists of ten statements, regarding students' classroom behaviours, observed over the previous 6-months, which are rated using a 3-point Likert scale. The items have been adapted from the Strengths and Difficulties questionnaire externalising subscale.

On-task behaviour. To determine the acute effect of the B2La intervention on students' behaviour in the classroom, observations will be conducted by trained research assistants at baseline and mid-intervention (3-months) using established methods [51]. The assessment includes a 30-minute observation period where research assistants will assess the on- and off-task behaviour of six randomly selected students (5 min per student). Observation and recording are completed in 15-sec intervals (20 observations per student) and teachers and students will not know who is being observed during the assessment.

For peer review only

Economic evaluation

We will assess the efficiency and affordability of the intervention using cost-effectiveness/cost utility analysis and budget impact respectively, conducted from a public finance perspective. The effectiveness measure will be based on the primary outcome (6MWT). Transformation of the CHUI-9D data will be employed in a cost utility analysis. The resource use and costs for the intervention and usual practice will be prospectively measured and derived from project records (staff and consumables), teacher surveys and school records. Additional costs in the intervention group are anticipated to be labour (implementation support), program development, and training costs. The cost effectiveness analysis will be conducted on a 'within trial' basis, that is, over the 6-month study period, comparing incremental costs and outcomes. Affordability of the program will be calculated using budget impact analysis, over a standard accounting cycle and is designed to assist decision making in schools and hence assist the translation of cost-effective and affordable programs. Scenario analysis will assess the costs to implement the program at scale across NSW. Reporting for the economic analysis will adhere to the CHEERS guidelines [52].

Process evaluation

We will conduct a process evaluation to determine program acceptability, implementation, adaptability, and sustainability in schools.

Acceptability: We will conduct focus groups to determine teachers' and participants' (i.e., students) perceptions of, and experiences with, the intervention. Teachers will also complete the Acceptability of Intervention Measure, Intervention Appropriateness Measure, and Feasibility of Intervention Measure [53].

Implementation: Teachers will be asked to record their delivery of B2La sessions using the teacher handbook. We will also track the number of sessions delivered using the B2La smartphone app. Members of our research team will conduct two session observations (using the SAAFE checklist) at each school to determine intervention fidelity. Finally, participants' mean heart rate during sessions will collected using the B2L app.

Adaptability: Teachers will be asked to reflect on how they adapted the intervention in the focus groups. This will include adaptions in relation to the characteristics of the school, class, and students.

For peer review only - http://bmjopen.bmj.com/site/about/guidelines.xhtml

Sustainability: Sustainability will be explored in the focus groups and via teacher and participant post-program evaluation questionnaires. Teachers will report their intention to deliver B2L in the future and complete an adapted version of the Program Sustainability Assessment Tool [54, 55]. Students will report their intention participate in HIIT in the two months following program completion.

Statistical analyses

Blinded analyses of the primary and secondary outcomes will be conducted by an independent statistician, using linear mixed models SAS V 9.1 (SAS Institute Inc, Cary, NC), with alpha levels set at $p<0.05$. The models will be used to assess the impact of group (B2La or control), time (treated as categorical with levels 6- and 9-months), and the group-by-time interaction. The models will include a random intercept for participant to account for the repeated measures for each participant, and a random intercept for school to account for the clustered design. The primary endpoint of the study will be 6-months from baseline. Least square mean differences between the treatment groups will be presented at both follow-up time points, with 95% confidence intervals and *p*-values. Compared to complete case analyses, mixed models include available data for all participants and are thus both more efficient and robust to bias. Mixed model analyses are consistent with the intention-to-treat principle, assuming the data are missing at random. The validity of this assumption will be explored by assessing relationships between missingness and observed values. We will conduct two sensitivity analyses for the primary outcome: (i) multiple imputation (assuming data are missing at random) and (ii) complete-case analysis (assuming data are missing completely at random). Four potential moderators (i.e., SES, sex, initial weight status and disability type) will be explored using interaction terms (i.e., time-by-treatment-by-moderator). If an interaction term is significant ($p<0.1$), sub-group analyses will be conducted.

DISCUSSION

Burn 2 Learn adapted has been designed to provide older adolescents with disability an opportunity to be active at school, but also focuses on developing their physical literacy (i.e., physical competence, confidence, knowledge, and motivation) to engage in vigorous physical activity. Importantly, our research team will provide teachers with training and support to ensure that the program is delivered in an engaging manner that supports students'

For peer review only - http://bmjopen.bmj.com/site/about/guidelines.xhtml

autonomous motivation to be active across the lifespan. Most HIIT studies have been delivered by researchers in controlled settings to establish efficacy, with little consideration of how they will work in the 'real world' [31]. By comparison, B2La was designed with scale-up in mind using the Consolidated Framework for Implementation Research to support implementation and sustainability. This may help to reduce the 'voltage drop' that typically occurs as interventions progress from efficacy to effectiveness to dissemination [29, 56-58].

ETHICS AND DISSEMINATION

Ethics approval for this cluster RCT was obtained from the Human Research Ethics Committee of the University of Newcastle, Australia (H-2021-0262) and the NSW Department of Education and Communities (SERAP:2021257). School Principals, teachers, parents, and students will all provide informed written consent prior to enrolment. It is not expected that participants will be at any greater risk of adverse events than they would be when participating in other types of school-based physical activity. However, the teacher handbook includes a section for teachers to report any injuries or adverse events that may occur. Any amendments to the study protocols will be publicly available via the Australian and New Zealand Clinical Trials Registry (Trial number: ACTRN12621000884808). Data management procedures will be conducted by DRL and AAL. All entered data will be de-identified using participant codes and will be stored electronically in a password protected drive at the University of Newcastle. The study will not involve a data monitoring committee, as the research team will be organised into evaluation (responsible for data collection and analysis) and implementation (responsible for intervention delivery and support).

Contributors: DRL, NS, CH, CL, NE, JJS, MN, PR, AAL, JB, PC secured funding for the project. All authors contributed intellectually to the study design and research methodology or will be directly involved in the collection and management of data. TK and DRL were responsible for drafting the manuscript. All authors provided critical review and endorsed the final version of the manuscript.

Competing interests: None declared.

Funding: The study is funded by Medical Research Future Fund (APP2007095). DRL is funded an National Health and Medical Research Council Senior Research Fellowship (APP1154507). This project is supported and co-designed with the NSW Department of

For peer review only

For peer review only - http://bmjopen.bmj.com/site/about/guidelines.xhtml

472 Education and Special Olympics Australia. The study funders will have no role in data collection, analysis, interpretation or writing. Nor will they influence over the publication of findings.

For peer review only

REFERENCES

1. World Health Organisation, *Towards a Common Language for Functioning, Disability and Health*. 2002, World Health Organization: Geneva.

2. World Health Organization, *World report on disability 2011*. 2011, World Health Organization: Geneva.

3. World Health Organization, *WHO global disability action plan 2014-2021: better health for all people with disability*. 2015, World Health Organization: Geneva.

4. Hassett, L., et al., *Comparisons of leisure-Time physical activity participation by adults with and without a disability: Results of an Australian cross-sectional national survey.* BMJ Open SEM, 2021. **7**: p. e000991.

5. McVilly, K., et al., *Diabetes in people with an intellectual disability: a systematic review of prevalence, incidence and impact.* Diabet Med, 2014. **31**(8): p. 897-904.

6. Rimmer, J.H., et al., *Obesity and obesity-related secondary conditions in adolescents with intellectual/developmental disabilities.* J Intellect Disabil Res, 2010. **54**(9): p. 787-94.

7. McKenzie, G., C. Willis, and N. Shields, *Barriers and facilitators of physical activity participation for young people and adults with childhood-onset physical disability: a mixed methods systematic review.* Dev Med Child Neurol, 2021. **63**(8): p. 914-924.

8. Shields, N. and A. Synnot, *Perceived barriers and facilitators to participation in physical activity for children with disability: a qualitative study.* BMC Pediatrics, 2016. **16**(1): p. 9.

9. Wright, A., et al., *Barriers and facilitators to physical activity participation for children with physical disability: comparing and contrasting the views of children, young people, and their clinicians.* Disabil Rehabil, 2019. **41**(13): p. 1499-1507.

For peer review only

For peer review only - http://bmjopen.bmj.com/site/about/guidelines.xhtml

499 10. McGarty, A.M., et al., *A systematic review and meta-analysis of interventions to increase physical activity in children and adolescents with intellectual disabilities.* J Intellect Disabil Res, 2018. **62**(4): p. 312-329.

502 11. Hills, A.P., D.R. Dengel, and D.R. Lubans, *Supporting public health priorities: recommendations for physical education and physical activity promotion in schools.* Prog Cardiovasc Dis, 2015. **57**(4): p. 368-74.

505 12. Rekaa, H., H. Hanisch, and B. Ytterhus, *Inclusion in Physical Education: Teacher Attitudes and Student Experiences. A Systematic Review.* Intl J Disabil Dev Educ, 2019. **66**(1): p. 36-55.

508 13. Haegele, J. and S. Sutherland, *Perspectives of Students with Disabilities Toward Physical Education: A Qualitative Inquiry Review.* Quest -Illinois- National Association for Physical Education in Higher Education-, 2015. **67**: p. 255-273.

511 14. Hardman, K., et al., *World-wide survey of school physical education.* 2013.

512 15. Naylor, P.J., et al., *Implementation of school based physical activity interventions: a systematic review.* Prev Med, 2015. **72**: p. 95-115.

514 16. Costigan, S., et al., *High-intensity interval training for improving health-related fitness in adolescents: A systematic review and meta-analysis.* BJSM, 2015. **49**.

516 17. Leahy, A., et al., *Review of High-Intensity Interval Training for Cognitive and Mental Health in Youth.* MSSE, 2020. **52**: p. 1.

518 18. Boer, P.H., et al., *The influence of sprint interval training on body composition, physical and metabolic fitness in adolescents and young adults with intellectual disability: a randomized controlled trial.* Clin Rehabil, 2014. **28**(3): p. 221-31.

521 19. Zwinkels, M., et al., *Effects of High-Intensity Interval Training on Fitness and Health in Youth With Physical Disabilities.* Pediatr Phys Ther, 2018. **31**: p. 1.

For peer review only - http://bmjopen.bmj.com/site/about/guidelines.xhtml

20. Leahy, A.A., et al., *School-based physical activity intervention for older adolescents: rationale and study protocol for the Burn 2 Learn cluster randomised controlled trial.* BMJ Open, 2019. **9**(5): p. e026029.

21. Leahy, A.A., et al., *Feasibility and Preliminary Efficacy of a Teacher-Facilitated High-Intensity Interval Training Intervention for Older Adolescents.* Pediatr Exerc Sci, 2019. **31**(1): p. 107-117.

22. Lubans, D., et al., *Time-efficient intervention to improve older adolescents' cardiorespiratory fitness: findings from the 'Burn 2 Learn' cluster randomised controlled trial.* BJSM, 2020. **55**: p. bjsports-2020.

23. Mavilidi, M.F., et al., *Effect of a Time-Efficient Physical Activity Intervention on Senior School Students' On-Task Behaviour and Subjective Vitality: the 'Burn 2 Learn' Cluster Randomised Controlled Trial.* Educ Psychol Rev, 2021. **33**(1): p. 299-323.

24. Leahy, A., et al., *Feasibility of a school-based physical activity intervention for adolescents with disability.* Pilot and Feasibility Studies, 2021. **7**.

25. Moher, D., et al., *CONSORT 2010 Explanation and Elaboration: updated guidelines for reporting parallel group randomised trials.* BMJ, 2010. **340**: p. c869.

26. McConkey, R. and J. Walsh, *An index of social competence for use in determining the service needs of mentally handicapped adults.* J Ment Defic Res, 1982. **26**(Pt 1): p. 47-61.

27. Elmahgoub, S.S., et al., *Reproducibility, validity and predictors of six-minute walk test in overweight and obese adolescents with intellectual disability.* Disabil Rehabil, 2012. **34**(10): p. 846-51.

For peer review only

For peer review only - http://bmjopen.bmj.com/site/about/guidelines.xhtml

28. Bartels, B., J.F. de Groot, and C.B. Terwee, *The six-minute walk test in chronic pediatric conditions: a systematic review of measurement properties.* Phys Ther, 2013. **93**(4): p. 529-41.

29. Beets, M.W., et al., *Identification and evaluation of risk of generalizability biases in pilot versus efficacy/effectiveness trials: a systematic review and meta-analysis.* IJBNPA, 2020. **17**(1): p. 19.

30. Australian Curriculum Assessment and Reporting Authority, *Guide to understanding the Index of Community Socioeducational Advantage (ICSEA)*, My School, Editor. 2020.

31. Lubans, D.R., et al., *Scaling-up Adolescent High-Intensity Interval Training Programs for Population Health.* Exerc Sport Sci Rev, 2022.

32. Beets, M.W., et al., *The theory of expanded, extended, and enhanced opportunities for youth physical activity promotion.* Int J Behav Nutr Phys Act, 2016. **13**(1): p. 120.

33. Ryan, R.M. and E.L. Deci, *Self-determination theory: Basic psychological needs in motivation, development, and wellness*. Self-determination theory: Basic psychological needs in motivation, development, and wellness. 2017, New York, NY, US: The Guilford Press. xii, 756-xii, 756.

34. Lubans, D.R., et al., *Framework for the design and delivery of organized physical activity sessions for children and adolescents: rationale and description of the 'SAAFE' teaching principles.* Int J Behav Nutr Phys Act, 2017. **14**(1): p. 24.

35. K, O.B., et al., *Self-determined motivation and physical activity in children and adolescents: a systematic review and meta-analysis.* Prev Med, 2014. **67**: p. 270-9.

36. Damschroder, L.J., et al., *Fostering implementation of health services research findings into practice: a consolidated framework for advancing implementation science.* Implement Sci, 2009. **4**: p. 50.

For peer review only

For peer review only - http://bmjopen.bmj.com/site/about/guidelines.xhtml

37. Shields, N., et al., *FitSkills: protocol for a stepped wedge cluster randomised trial of a community-based exercise programme to increase participation among young people with disability.* BMJ Open, 2020. **10**(7): p. e037153.

38. Wouters, M., H.M. Evenhuis, and T.I. Hilgenkamp, *Systematic review of field-based physical fitness tests for children and adolescents with intellectual disabilities.* Res Dev Disabil, 2017. **61**: p. 77-94.

39. Damen, K.M.S., et al., *6-Minute Push Test in Youth Who Have Spina Bifida and Who Self-Propel a Wheelchair: Reliability and Physiologic Response.* Phys Ther, 2020. **100**(10): p. 1852-1861.

40. Boer, P.H. and S.J. Moss, *Test-retest reliability and minimal detectable change scores of twelve functional fitness tests in adults with Down syndrome.* Res Dev Disabil, 2016. **48**: p. 176-85.

41. Winnick, J.P. and F.X. Short, *Brockport Physical Fitness Test Manual : A Health-Related Assessment for Youngsters With Disabilities*, in *Brockport Physical Fitness Test Manual : A Health-Related Assessment for Youngsters With Disabilities*, J.P. Winnick and F.X. Short, Editors. 2014, Human Kinetics: Champaign, IL.

42. Cole, T.J. and T. Lobstein, *Extended international (IOTF) body mass index cut-offs for thinness, overweight and obesity.* Pediatr Obes, 2012. **7**(4): p. 284-94.

43. Weintraub, S., et al., *Cognition assessment using the NIH Toolbox.* Neurology, 2013. **80**(11 Suppl 3): p. S54-64.

44. Hessl, D., et al., *The NIH Toolbox Cognitive Battery for intellectual disabilities: three preliminary studies and future directions.* J Neurodev Disord, 2016. **8**(1): p. 35.

45. Stevens, K. and J. Ratcliffe, *Measuring and valuing health benefits for economic evaluation in adolescence: an assessment of the practicality and validity of the child*

For peer review only

For peer review only - http://bmjopen.bmj.com/site/about/guidelines.xhtml

health utility 9D in the Australian adolescent population. Value Health, 2012. **15**(8): p. 1092-9.

46. Markland, D. and V. Tobin, *A Modification to the Behavioural Regulation in Exercise Questionnaire to Include an Assessment of Amotivation.* JSEP, 2004. **26**(2): p. 191-196.

47. Eather, N., et al., *Development and Evaluation of the High-Intensity Interval Training Self-Efficacy Questionnaire.* J Sport Exerc Psychol, 2020: p. 1-9.

48. Lubans, D.R., et al., *Development, test-retest reliability, and construct validity of the resistance training skills battery.* J Strength Cond Res, 2014. **28**(5): p. 1373-80.

49. Bebich-Philip, M.D., et al., *Adaptation of the Resistance Training Skills Battery for Use in Children Across the Motor Proficiency Spectrum.* Pediatr Exerc Sci, 2016. **28**(3): p. 473-80.

50. Mellor, D., *Normative data for the Strengths and Difficulties Questionnaire in Australia.* Aust Psychol, 2005. **40**: p. 215-222.

51. Alerto, P.A. and A.C. Troutman, *Applied Behavior Analysis for Teachers*. 2003, Pearson: Australia.

52. Husereau, D., et al., *Consolidated Health Economic Evaluation Reporting Standards 2022 (CHEERS 2022) statement: updated reporting guidance for health economic evaluations.* BJOG, 2022. **129**(3): p. 336-344.

53. Weiner, B.J., et al., *Psychometric assessment of three newly developed implementation outcome measures.* Implement Sci, 2017. **12**(1): p. 108.

54. Hall, A., et al., *Adaptation and Validation of the Program Sustainability Assessment Tool (PSAT) for Use in the Elementary School Setting.* Int J Environ Res Public Health, 2021. **18**(21).

For peer review only

55. Luke, D., et al., *The Program Sustainability Assessment Tool: A New Instrument for Public Health Programs.* Prev. Chronic Dis., 2014. **11**: p. E12.

56. Chambers, D., R. Glasgow, and K. Stange, *The dynamic sustainability framework: Addressing the paradox of sustainment amid ongoing change.* Implementation science : IS, 2013. **8**: p. 117.

57. Lane, C., et al., *How effective are physical activity interventions when they are scaled-up: a systematic review.* Int J Behav Nutr Phys Act, 2021. **18**(1): p. 16.

58. McCrabb, S., et al., *Scaling-up evidence-based obesity interventions: A systematic review assessing intervention adaptations and effectiveness and quantifying the scale-up penalty.* Obes Rev, 2019. **20**(7): p. 964-982.

For peer review only

For peer review only - http://bmjopen.bmj.com/site/about/guidelines.xhtml

For peer review only

For peer review only - http://bmjopen.bmj.com/site/about/guidelines.xhtml

Figure 1: Example B2La HIIT technique card

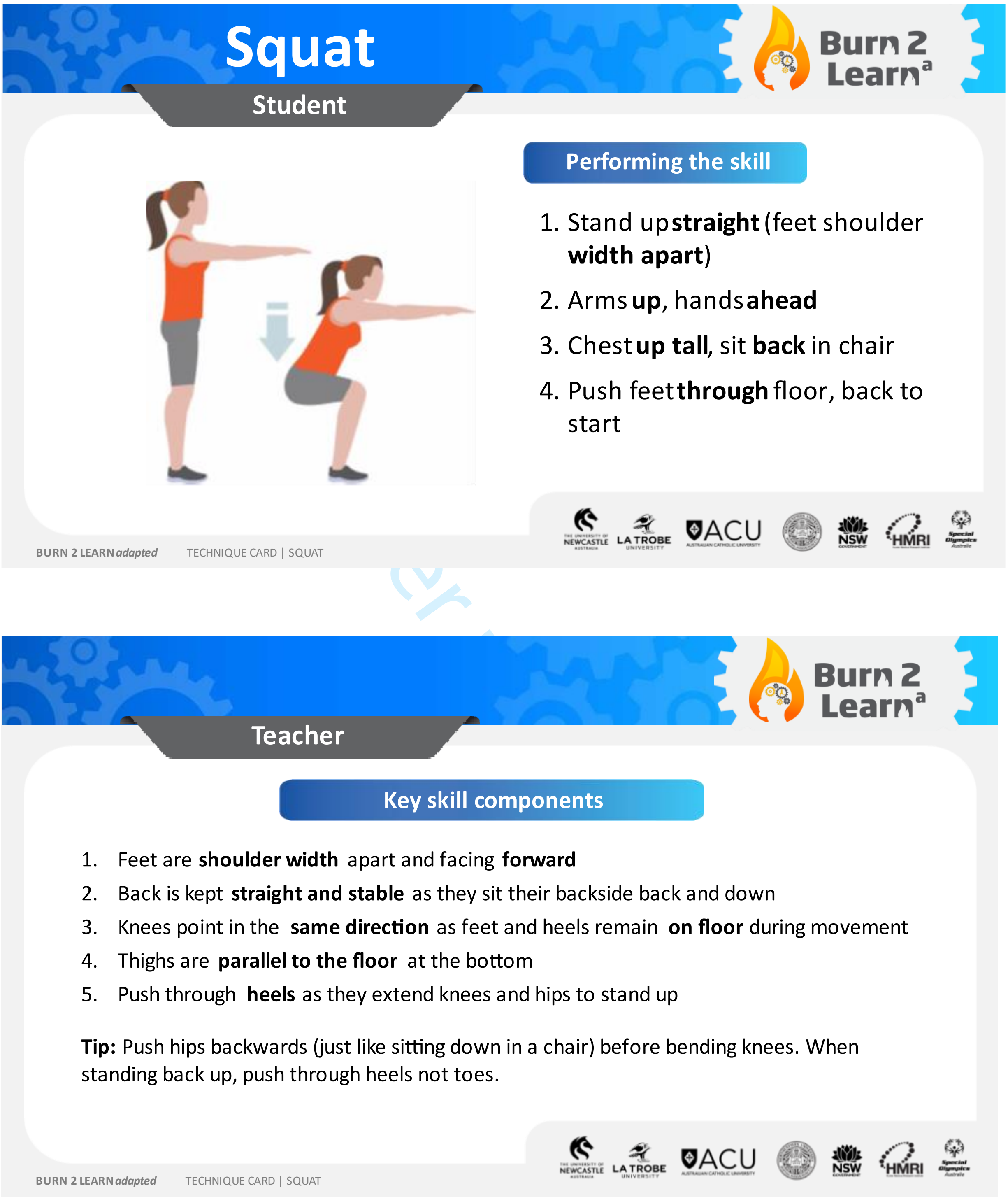

Figure 2: Example B2La HIIT task cards

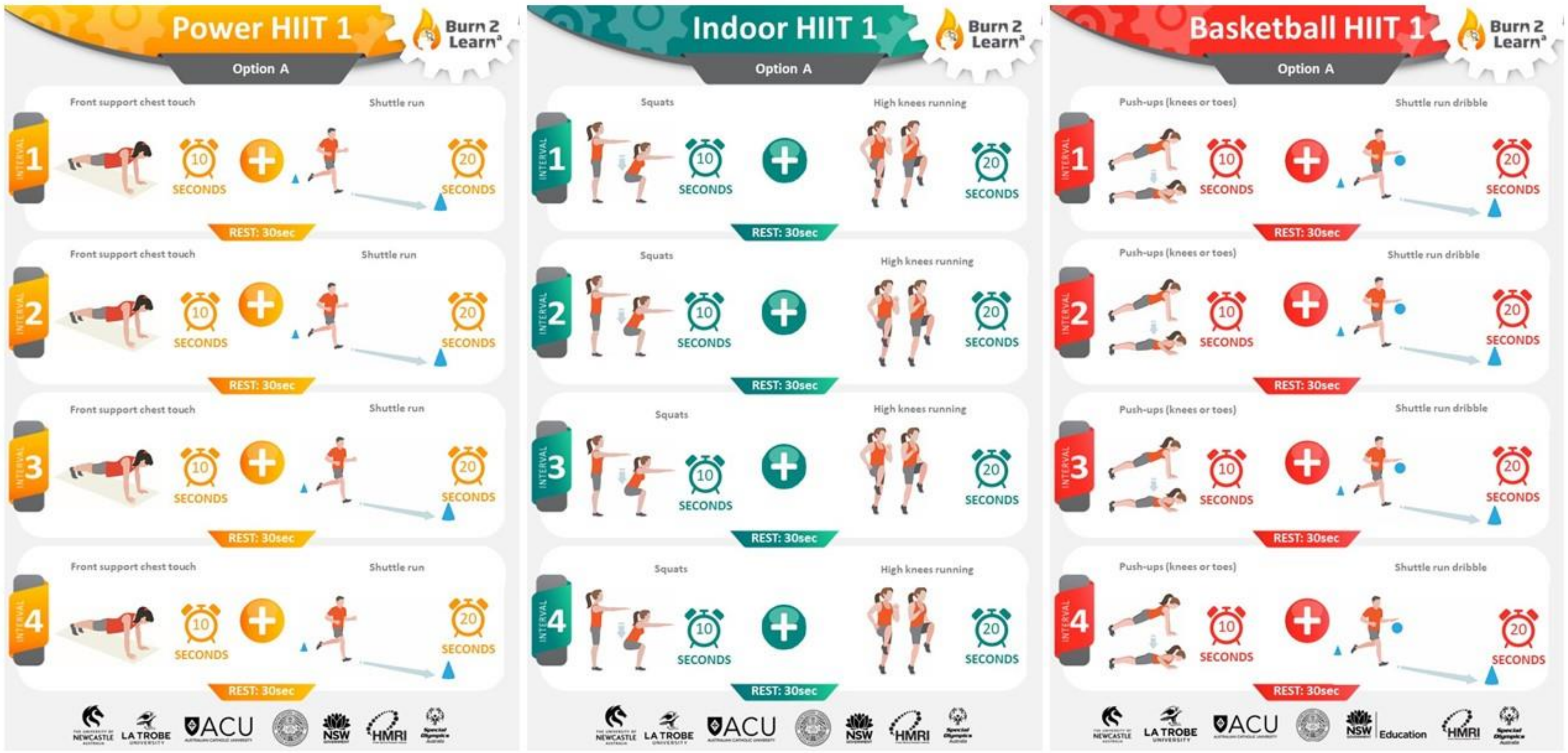

For peer review only

STANDARD PROTOCOL ITEMS: RECOMMENDATIONS FOR INTERVENTIONAL TRIALS

SPIRIT 2013 Checklist: Recommended items to address in a clinical trial protocol and related documents*

Section/item	Item No	Description	Addressed on page number
**Administrative information**			
Title	1	Descriptive title identifying the study design, population, interventions, and, if applicable, trial acronym	_1___________
Trial registration	2a	Trial identifier and registry name. If not yet registered, name of intended registry	_3___________
	2b	All items from the World Health Organization Trial Registration Data Set	__NA_________
Protocol version	3	Date and version identifier	__7__________
Funding	4	Sources and types of financial, material, and other support	_18__________
Roles and responsibilities	5a	Names, affiliations, and roles of protocol contributors	_1___________
	5b	Name and contact information for the trial sponsor	_18__________
	5c	Role of study sponsor and funders, if any, in study design; collection, management, analysis, and interpretation of data; writing of the report; and the decision to submit the report for publication, including whether they will have ultimate authority over any of these activities	_____18_______
	5d	Composition, roles, and responsibilities of the coordinating centre, steering committee, endpoint adjudication committee, data management team, and other individuals or groups overseeing the trial, if applicable (see Item 21a for data monitoring committee)	__NA________

For peer review only

Introduction

Background and rationale	6a	Description of research question and justification for undertaking the trial, including summary of relevant studies (published and unpublished) examining benefits and harms for each intervention	_5-6_________
	6b	Explanation for choice of comparators	____9_________
Objectives	7	Specific objectives or hypotheses	_7___________
Trial design	8	Description of trial design including type of trial (eg, parallel group, crossover, factorial, single group), allocation ratio, and framework (eg, superiority, equivalence, noninferiority, exploratory)	_7___________

Methods: Participants, interventions, and outcomes

Study setting	9	Description of study settings (eg, community clinic, academic hospital) and list of countries where data will be collected. Reference to where list of study sites can be obtained	_8___________
Eligibility criteria	10	Inclusion and exclusion criteria for participants. If applicable, eligibility criteria for study centres and individuals who will perform the interventions (eg, surgeons, psychotherapists)	_8___________
Interventions	11a	Interventions for each group with sufficient detail to allow replication, including how and when they will be administered	_10-12_______
	11b	Criteria for discontinuing or modifying allocated interventions for a given trial participant (eg, drug dose change in response to harms, participant request, or improving/worsening disease)	__NA_________
	11c	Strategies to improve adherence to intervention protocols, and any procedures for monitoring adherence (eg, drug tablet return, laboratory tests)	______13______
	11d	Relevant concomitant care and interventions that are permitted or prohibited during the trial	______NA______
Outcomes	12	Primary, secondary, and other outcomes, including the specific measurement variable (eg, systolic blood pressure), analysis metric (eg, change from baseline, final value, time to event), method of aggregation (eg, median, proportion), and time point for each outcome. Explanation of the clinical relevance of chosen efficacy and harm outcomes is strongly recommended	_14-15_______
Participant timeline	13	Time schedule of enrolment, interventions (including any run-ins and washouts), assessments, and visits for participants. A schematic diagram is highly recommended (see Figure)	_7___________

For peer review only - http://bmjopen.bmj.com/site/about/guidelines.xhtml

Sample size	14	Estimated number of participants needed to achieve study objectives and how it was determined, including clinical and statistical assumptions supporting any sample size calculations	_8-9_________
Recruitment	15	Strategies for achieving adequate participant enrolment to reach target sample size	_8___________
**Methods: Assignment of interventions (for controlled trials)**			
Allocation:			
Sequence generation	16a	Method of generating the allocation sequence (eg, computer-generated random numbers), and list of any factors for stratification. To reduce predictability of a random sequence, details of any planned restriction (eg, blocking) should be provided in a separate document that is unavailable to those who enrol participants or assign interventions	_9___________
Allocation concealment mechanism	16b	Mechanism of implementing the allocation sequence (eg, central telephone; sequentially numbered, opaque, sealed envelopes), describing any steps to conceal the sequence until interventions are assigned	_9___________
Implementation	16c	Who will generate the allocation sequence, who will enrol participants, and who will assign participants to interventions	_9___________
Blinding (masking)	17a	Who will be blinded after assignment to interventions (eg, trial participants, care providers, outcome assessors, data analysts), and how	_9___________
	17b	If blinded, circumstances under which unblinding is permissible, and procedure for revealing a participant's allocated intervention during the trial	____13-14____
**Methods: Data collection, management, and analysis**			
Data collection methods	18a	Plans for assessment and collection of outcome, baseline, and other trial data, including any related processes to promote data quality (eg, duplicate measurements, training of assessors) and a description of study instruments (eg, questionnaires, laboratory tests) along with their reliability and validity, if known. Reference to where data collection forms can be found, if not in the protocol	_13-15______
	18b	Plans to promote participant retention and complete follow-up, including list of any outcome data to be collected for participants who discontinue or deviate from intervention protocols	__14_________

Data management	19	Plans for data entry, coding, security, and storage, including any related processes to promote data quality (eg, double data entry; range checks for data values). Reference to where details of data management procedures can be found, if not in the protocol	_18________
Statistical methods	20a	Statistical methods for analysing primary and secondary outcomes. Reference to where other details of the statistical analysis plan can be found, if not in the protocol	_17________
	20b	Methods for any additional analyses (eg, subgroup and adjusted analyses)	_17__________
	20c	Definition of analysis population relating to protocol non-adherence (eg, as randomised analysis), and any statistical methods to handle missing data (eg, multiple imputation)	_17________
**Methods: Monitoring**			
Data monitoring	21a	Composition of data monitoring committee (DMC); summary of its role and reporting structure; statement of whether it is independent from the sponsor and competing interests; and reference to where further details about its charter can be found, if not in the protocol. Alternatively, an explanation of why a DMC is not needed	____18_______
	21b	Description of any interim analyses and stopping guidelines, including who will have access to these interim results and make the final decision to terminate the trial	_____NA_____
Harms	22	Plans for collecting, assessing, reporting, and managing solicited and spontaneously reported adverse events and other unintended effects of trial interventions or trial conduct	_18__________
Auditing	23	Frequency and procedures for auditing trial conduct, if any, and whether the process will be independent from investigators and the sponsor	_____NA_____
**Ethics and dissemination**			
Research ethics approval	24	Plans for seeking research ethics committee/institutional review board (REC/IRB) approval	_18________
Protocol amendments	25	Plans for communicating important protocol modifications (eg, changes to eligibility criteria, outcomes, analyses) to relevant parties (eg, investigators, REC/IRBs, trial participants, trial registries, journals, regulators)	_18________

For peer review only - http://bmjopen.bmj.com/site/about/guidelines.xhtml

Consent or assent	26a	Who will obtain informed consent or assent from potential trial participants or authorised surrogates, and how (see Item 32)	_18_________
	26b	Additional consent provisions for collection and use of participant data and biological specimens in ancillary studies, if applicable	_____NA______
Confidentiality	27	How personal information about potential and enrolled participants will be collected, shared, and maintained in order to protect confidentiality before, during, and after the trial	_18___________
Declaration of interests	28	Financial and other competing interests for principal investigators for the overall trial and each study site	_18___________
Access to data	29	Statement of who will have access to the final trial dataset, and disclosure of contractual agreements that limit such access for investigators	_______18_______
Ancillary and post-trial care	30	Provisions, if any, for ancillary and post-trial care, and for compensation to those who suffer harm from trial participation	________18______
Dissemination policy	31a	Plans for investigators and sponsor to communicate trial results to participants, healthcare professionals, the public, and other relevant groups (eg, via publication, reporting in results databases, or other data sharing arrangements), including any publication restrictions	_____18__________
	31b	Authorship eligibility guidelines and any intended use of professional writers	_18___________
	31c	Plans, if any, for granting public access to the full protocol, participant-level dataset, and statistical code	__NA________
**Appendices**			
Informed consent materials	32	Model consent form and other related documentation given to participants and authorised surrogates	_Yes__________
Biological specimens	33	Plans for collection, laboratory evaluation, and storage of biological specimens for genetic or molecular analysis in the current trial and for future use in ancillary studies, if applicable	_____NA______

*It is strongly recommended that this checklist be read in conjunction with the SPIRIT 2013 Explanation & Elaboration for important clarification on the items. Amendments to the protocol should be tracked and dated. The SPIRIT checklist is copyrighted by the SPIRIT Group under the Creative Commons "Attribution-NonCommercial-NoDerivs 3.0 Unported" license.

Time-efficient physical activity intervention for older adolescents with disability: Rationale and study protocol for the Burn 2 Learn adapted (B2La) cluster randomised controlled trial

Journal:	*BMJ Open*
Manuscript ID	bmjopen-2022-065321.R1
Article Type:	Protocol
Date Submitted by the Author:	13-Jul-2022
Complete List of Authors:	Kable, Toby; The University of Newcastle, School of Education; The University of Newcastle Hunter Medical Research Institute<br>Leahy, Angus; The University of Newcastle, PRC for Physical Activity and Nutrition; The University of Newcastle Hunter Medical Research Institute<br>Smith, Jordan ; The University of Newcastle, School of Education; The University of Newcastle Hunter Medical Research Institute<br>Eather, Narelle; The University of Newcastle, School of Education; The University of Newcastle Hunter Medical Research Institute<br>Shields, Nora; La Trobe University,<br>Noetel, Michael; Australian Catholic University - Blacktown Campus, Institute for Positive Psychology and Education; Australian Catholic University - Blacktown Campus, School of Behavioural and Health Sciences<br>Lonsdale, Chris; Australian Catholic University - Blacktown Campus<br>Hillman, Charles; Northeastern University, Department of Psychology; Northeastern University, Department of Physical Therapy<br>Reeves, Penny; University of Newcastle Hunter Medical Research Institute, Health Research Economics; The University of Newcastle, School of Medicine and Public Health<br>Oldmeadow, Christopher; The University of Newcastle Hunter Medical Research Institute<br>Kennedy, Sarah; Western Sydney University, School of Health Sciences<br>Boyer, James; NSW Department of Education, School Sport Unit<br>Stimpson, Leisl; Special Olympics Australia, Education & Training<br>Comis, Pierre; Special Olympics Australia<br>Roche, Laura; The University of Newcastle, School of Education<br>Lubans, David; The University of Newcastle, School of Education
**Primary Subject Heading**:	Sports and exercise medicine
Secondary Subject Heading:	Public health
Keywords:	PUBLIC HEALTH, SPORTS MEDICINE, STATISTICS & RESEARCH METHODS

SCHOLARONE™
Manuscripts

Time-efficient physical activity intervention for older adolescents with disability: Rationale and study protocol for the Burn 2 Learn adapted (B2La) cluster randomised controlled trial

Toby Kable[1,2], Angus A. Leahy[1,2], Jordan J. Smith[1,2], Narelle Eather[1,2], Nora Shields[3], Michael Noetel[4], Chris Lonsdale[5], Charles H. Hillman[6,7], Penny Reeves[8,2], Christopher Oldmeadow[8,2], Sarah G. Kennedy[9], James Boyer[10], Leisl Stimpson[11], Pierre Comis[11], Laura Roche[12], David R. Lubans§[1,2]

[1] Centre for Active Living and Learning, College of Human and Social Futures, University of Newcastle, Callaghan, New South Wales, Australia

[2] Hunter Medical Research Institute, New Lambton Heights, NSW, Australia

[3] Department of Physiotherapy, Podiatry and Prosthetics and Orthotics, La Trobe University, Melbourne, Victoria, Australia

[4] School of Behavioural and Health Sciences, Faculty of Health Sciences, Australian Catholic University, Banyo, New South Wales, Australia

[5] Institute for Positive Psychology and Education, Faculty of Health Sciences, Australian Catholic University, North Sydney, New South Wales, Australia

[6] Department of Psychology, Northeastern University, Boston, Massachusetts, United States of America

[7] Department of Physical Therapy, Movement and Rehabilitation Sciences, Northeastern University, Boston, Massachusetts, United States of America

[8] School of Medicine and Public Health, College of Health, Medicine and Wellbeing, University of Newcastle, Callaghan, New South Wales, Australia

[9]School of Health Sciences, Translational Health Research Institute, Western Sydney University, Darug Country, New South Wales, Australia

[10] New South Wales Department of Education, Turrella, New South Wales, Australia

[11] Special Olympics Australia, Concord West, New South Wales, Australia

[12] Special and Inclusive Education, School of Education, College of Human and Social Futures, University of Newcastle, Callaghan, New South Wales, Australia

For peer review only - http://bmjopen.bmj.com/site/about/guidelines.xhtml

§Corresponding author details
Professor David Lubans
Centre for Active Living and Learning
School of Education, University of Newcastle
Callaghan, NSW, Australia 2308
P: +612 4921 2049
E: David.Lubans@newcastle.edu.au

Key words: Exercise; High-intensity interval training; Adolescents; Disability; Special Education; Behaviour change; Resistance Training; Mental health; Cognition

Word count: 4,900 (1 table, and 2 figures)

For peer review only

ABSTRACT

Introduction: Physical activity declines during adolescence, with the lowest levels of activity observed among those with disability. Schools are ideal settings to address this issue; however, few school-based interventions have been specifically designed for older adolescents with disability. Our aim is to investigate the effects of a school-based physical activity program, involving high-intensity interval training (HIIT), on physical, mental, and cognitive health in older adolescents with disability.

Methods and Analysis: We will evaluate the Burn 2 Learn *adapted* (B2La) intervention using a two-arm, parallel group, cluster randomised controlled trial with allocation occurring at the school level (treatment or wait-list control). Secondary schools will be recruited in two cohorts from New South Wales, Australia. We will aim to recruit 300 older adolescents (aged 15-19 years) with disability from 30 secondary schools (10 in Cohort 1 and 20 in Cohort 2). Schools allocated to the intervention group will deliver two HIIT sessions per week during scheduled specialist support classes. The sessions will include foundational aerobic and muscle strengthening exercises tailored to meet student needs. We will provide teachers with training, resources, and support to facilitate the delivery of the B2La program. Study outcomes will be assessed at baseline, 6-months (primary endpoint) and 9-months. Our primary outcome is functional capacity assessed using the 6-Minute Walk/Push Test. Secondary outcomes include physical activity, muscular fitness, body composition, cognitive function, quality of life, physical literacy, and on-task behaviour in the classroom. We will also conduct economic and process evaluations to determine cost effectiveness, program acceptability, implementation, adaptability, and sustainability in schools.

Ethics and dissemination: This study has received approval from the University of Newcastle (H-2021-0262) and the New South Wales (NSW) Department of Education (SERAP: 2021257) human research ethics committees. Findings will be published in peer-reviewed journals, and key stakeholders will be provided with a detailed report following the study.

Trial registration: Australian New Zealand Clinical Trials Registry Number: ACTRN12621000884808.

For peer review only - http://bmjopen.bmj.com/site/about/guidelines.xhtml

Strengths and limitations of this study

Strengths of this study include:

- Our cluster randomised controlled trial will be adequately powered to detect meaningful changes in the primary outcome.
- Informed by our pilot work, we have tailored the intervention and assessment processes to increase accessibility for the unique study population.
- The Burn 2 Learn adapted intervention has been designed in consultation with adolescents with disability, and key stakeholders (i.e., NSW Department of Education and Special Olympics Australia).

Limitations of the study include:

- Having a unique study population with physical and/or intellectual limitations, not all participants will be able to complete all measures.
- It might not be possible to blind assessors for all outcomes, as group allocation is often revealed by research participants and teachers during post-test assessments in school-based trials.

For peer review only

INTRODUCTION

Disability is an umbrella term used to describe impairments (i.e., problems in body function or structure), activity limitations (i.e., difficulty in performing activities), and participation restrictions (i.e., difficulty engaging in life situations) [1]. Disability is a worldwide public health and human rights issue with 15% of the global population estimated to be living with disability [2]. These individuals often face widespread barriers to accessing health and related services, and have poorer health outcomes than those without disability [3]. As noted in the WHO's Global Disability Action Plan, the burden of disability can be reduced by addressing the determinants of health, including participation in physical activity [3]. Individuals with disability are typically less physically active [4] and more likely to have co-occurring chronic and complex lifestyle diseases [5, 6] than those without disability.

Young people with disability face many common and unique barriers to participation in physical activity. Previous research has identified personal (i.e., injury, lack of skills and time to exercise), social (i.e., unsupportive peers and parents), and environmental (i.e., inadequate accessibility and lack of appropriate programs) barriers to participation in this population [7, 8]. Conversely, factors shown to facilitate participation include having the time available, being involved in programs that are adaptable, and exercising in a group with people of a similar age [9]. Parents also play a critical role in determining whether young people with disability are physically active [8]. Although parents typically acknowledge the value of physical activity and want their children to be active, many also express concerns about time commitments, balancing the needs of family members, and the suitability of programs [8]. To date, the majority of physical activity interventions targeting young people with disability have been conducted in clinical, community, and home settings [10].

Schools are ideal for physical activity promotion, as they provide access to the adolescent population and have the necessary equipment, facilities, and personnel to deliver programs [11]. Physical education (PE) is the primary means of physical activity promotion in schools, and there is a large body of research focusing on the inclusion of children and adolescents with disability in PE classes [12]. Although teachers typically advocate for inclusion in PE, many lack the confidence and competence to successfully involve students with disability in ways that truly benefit their physical literacy [12]. Moreover, students with disability often feel marginalised when participating in mainstream PE classes, commonly reporting feelings of social isolation, bullying, and negative social comparisons [13]. Regardless, simply

For peer review only - http://bmjopen.bmj.com/site/about/guidelines.xhtml

integrating students with disability into mainstream PE is not enough to produce meaningful changes in health, particularly in the final years of school where there is no mandatory physical activity for students [14]. In light of this, there is a need for innovative school-based physical activity interventions designed specifically for older adolescents with disability.

Implementing health promotion interventions with older adolescents is challenging, and lack of time is a major barrier to physical activity promotion for this age group [15]. For older adolescents with disability, the final years of school also involves participation in programs facilitating transition into post school pathways (e.g., community access and transition to work programs). Given that adolescents with disability have many competing needs as they prepare for life after schooling, school-based physical activity programs will have the best chances of adoption if they do not require a substantial time commitment. However, physical activity programs that provide only a small 'dose' of activity are also unlikely to have meaningful health benefits; that is, unless the physical activity offered is of high intensity.

High-intensity interval training (HIIT) is a time-efficient strategy for improving physical, mental, and cognitive health in typically developing adolescents [16, 17]. HIIT sessions generally consist of several short bouts of vigorous activity interspersed with brief periods of light activity or rest. HIIT allows participants to experience similar benefits to other modes of exercise, in less time. Previous studies evaluating school-based HIIT programs for adolescents with disability have shown improvements in physical health (i.e., body composition, aerobic fitness), but have been delivered by physiotherapists or experienced physical educators [18, 19]. Such programs have limited scalability due to ongoing costs required for intervention implementation. To enhance program scalability, having classroom teachers implement the intervention has greater potential to change school practice.

We recently conducted a large-scale evaluation of the first teacher-facilitated school-based HIIT intervention, known as Burn 2 Learn (B2L), for older adolescents in mainstream schools [20-23]. Briefly, teachers were trained to deliver 2-3 HIIT sessions per week for 16 weeks during students' regular academic lessons. Positive effects were observed for the primary outcome of cardio-respiratory fitness (CRF), as well as a range of secondary outcomes (e.g., muscular fitness, mental health, and classroom engagement). Following the success of the intervention, our research team was approached by a local school to adapt the B2L intervention for students with disability. We subsequently conducted a pilot study of the

For peer review only - http://bmjopen.bmj.com/site/about/guidelines.xhtml

Burn 2 Learn adapted (B2La) program in one secondary school [24]. We found it was feasible to train special and inclusive education teachers to deliver the B2La sessions, which were well received by teachers and students. We also found preliminary support for program efficacy for improving functional capacity and muscular fitness. Following our successful feasibility study, we partnered with the NSW Department of Education and Special Olympics Australia to refine and evaluate B2La in a larger effectiveness trial.

Study objectives

The primary aim of this trial is to determine the effect of the B2La intervention on functional capacity (primary outcome) in older adolescents with disability. Secondary outcomes include physical activity (accelerometers), muscular fitness, body composition, cognitive function, mental health, physical literacy, and on-task classroom behaviour. We will also conduct a detailed economic and process evaluation to determine cost effectiveness, program efficiency, acceptability, implementation, adaptability, and practicality.

METHODS

Study design

Our trial is registered with the Australian New Zealand Clinical Trials Registry (ACTRN12621000884808). The reporting of this trial will adhere to the CONSORT [25] guidelines. The B2La intervention will be evaluated using a two-arm parallel group cluster randomised controlled trial (RCT) with a treatment group and wait-list control group. Assessments will occur at baseline, 6- (primary endpoint) and 9-months from baseline (secondary endpoint). The RCT will be conducted with two cohorts, one starting in 2022 (10 schools; 5 intervention and 5 control), and another starting in 2023 (20 schools; 10 intervention and 10 control). Baseline data collection will occur in the school term preceding intervention delivery (i.e., Term 1 [February to April 2022 and 2023]. The intervention will be delivered during Terms 2, 3 and 4 [April to November 2022 and 2023]. Immediate post-intervention data collection (i.e., ~6-months) will occur at the end of Term 3 (August to September 2022 and 2023), and follow-up assessments (i.e., ~9-months) will be completed in Term 4 (November to December 2022 and 2023).

School recruitment and selection

NSW Government, Catholic, and Independent secondary schools with student cohorts that include older adolescents (i.e., Grades 10 to 12, students aged 15-19) with disability will be

For peer review only - http://bmjopen.bmj.com/site/about/guidelines.xhtml

eligible to participate. Schools will include both mainstream schools with specialist support classes and Schools for Specific Purposes (SSPs). SSPs are dedicated schools for students with moderate-to-high learning and support needs. Eligible schools will be identified, and an expression of interest directed to the school principal. Interested schools will then liaise with the project manager to address any questions or concerns they have prior to returning informed consent.

Participants

Students at the study schools will be eligible to participate if they are: (i) in Grades 10 to 12 (15-19 years) and identify as living with disability (including neurodevelopmental disability, physical, intellectual, or sensory disabilities), (ii) able to follow simple verbal instructions in English (as determined by the Index of Social Competence) [26], and (iii) able to participate in vigorous intensity exercise (wheelchair users will be eligible). We will aim to recruit 10 students from each school. We will also recruit two special and inclusive education teachers per school, who will act as school champions and facilitate the delivery of B2La sessions. Special and inclusive education teachers develop and deliver specialised learning programs for students with a range of disabilities and learning difficulties.

Sample size and power calculation

Power calculations were based on the primary outcome of functional capacity, assessed using the 6-Minute Walk Test (6MWT), which has good reliability in adolescents with disability (ICC =0.82) [27]. A 6-Minute Push Test will be administered for wheelchair users. Although adolescent data are lacking, studies conducted among adult populations with chronic health conditions have reported minimal clinically important differences (MCID) of 24 to 44 metres using the 6MWT [28]. In our pilot study, we observed a large increase in distance covered from baseline to immediately after the intervention period (163 ± 131 m). However, our pilot study did not include a control group and effects are typically smaller in effectiveness trials compared with pilot studies [29]. Based on the pilot data, we estimate a treatment effect of 80m will represent a MCID in our population. Through simulations (n=10,000) and using data from our pilot study (i.e., baseline post-test correlation of r = 0.60, standard deviation of 90m and intraclass correlation of 0.2), we have determined we will require a sample of 30 schools with 7 participants per school. This sample size will be enough to detect a MCID of 80m with 90% power at a 5% significance level. Allowing for 30% loss to follow-up at 6-months we will aim to recruit 10 students from 30 schools (total sample size of 300).

For peer review only - http://bmjopen.bmj.com/site/about/guidelines.xhtml

Blinding and randomisation

Randomisation will occur within each cohort once consenting schools have completed baseline assessments. Schools will be matched as closely as possible based on the following characteristics in this order: (i) school type (i.e., mainstream school support class/SSP), (ii) school sector (i.e., Government/Catholic/Independent), (iii) geographic location (i.e., region, rural/urban, coastal/inland), and (iv) student population educational advantage (i.e., using the Index of Community Socio-Educational Advantage [ICSEA]) [30]. Matched schools will be randomised to the intervention or wait-list control group using a random number producing algorithm by an independent statistical analysis service – Clinical Research Design, Information Technology and Statistical Support (CREDITSS) run by the Hunter Medical Research Institute. One school from each pair will be allocated to the B2La condition and the other to the wait-list control condition. Schools randomised to the intervention group will deliver the B2La program during the study period, whereas schools allocated to the wait-list control group will continue with usual school practice (i.e., normal curricular lessons) for the duration of the study period and will receive the intervention the following year. We decided to use a wait-list control design, rather than an attention-matched placebo because: (i) our research team will have minimal contact with students, and (ii) our findings will have greater external validity, as participants in the control group will receive 'usual practice'.

Patient and public involvement

Following the B2L cluster RCT, our research team was asked to adapt the intervention for students with disability. We subsequently conducted a feasibility study in one secondary school in Newcastle (N = 16 students) [24]. Participating students and teachers were invited to provide feedback on the intervention and suggestions for further improvement. This feedback was then used to refine intervention components (e.g., exercise sessions) and develop implementation strategies (e.g., professional learning for teachers). We then partnered with the NSW Department of Education and Special Olympics Australia to create B2La. We conducted further testing with teachers and students with disability before progressing to this trial.

Intervention

Intervention delivery

The B2La intervention will be delivered in four phases:

For peer review only - http://bmjopen.bmj.com/site/about/guidelines.xhtml

1. Laying the foundation (weeks 1 to 4)
2. Developing a routine (weeks 5 to 9),
3. Maintaining student interest (weeks 10 to 16), and
4. Moving towards independence (week 17 onwards).

In Phases 1-3 (16 weeks), teachers will facilitate the delivery of at least two HIIT sessions/week during lesson-time. Phase 1 will start with a 4-week block to familiarise students with the B2La session structure and support resources, and to develop the foundational exercise skills that are used within the HIIT sessions utilising the B2La technique cards (Figure 1). During this phase, students will participate in two HIIT workouts: Indoor HIIT and Power HIIT (as shown in Figure 2). These basic HIIT workouts do not require additional sport equipment or partner interaction. This will allow students to develop their movement skill competency.

In Phases 2 and 3 (weeks 5 to 16) the number of foundational exercises used within the HIIT sessions will increase as students become more confident with the exercises and session routine. During this phase there will be an increase in the work interval within the HIIT sessions. Students will also be introduced to novel HIIT themed workouts, including: Soccer HIIT and Basketball HIIT (Phase 2), and Judo HIIT, Cricket HIIT and Custom HIIT (Phase 3). During Phases 2 and 3, teachers will be encouraged to increase the amount of autonomy provided to students (e.g., choice of B2La session). In Phase 4 (week 17 onwards), students will be encouraged to engage in physical activity sessions of interest outside of school (e.g., at home, the park), but teachers may continue to facilitate B2La sessions during lessons if they choose. Students will have their own B2La goal setting activity booklet.

For peer review only - http://bmjopen.bmj.com/site/about/guidelines.xhtml

Intervention components

The B2La intervention includes the following components: student information seminar, school-based HIIT sessions, smartphone application (app), goal setting activity booklet, and parental support videos. *Student information seminar:* Delivered by teachers, the seminar will provide an overview of B2La and focus on the barriers and benefits of physical activity for adolescents with disability [4] as well as evidence-based behaviour change techniques (e.g., self-monitoring, self-assessment, and goal setting). Teachers will be provided with a PowerPoint presentation template with embedded videos developed specifically for this project.

School-based exercise sessions: Sessions will be delivered during scheduled 'Learning Support Lessons'; a time-period when adolescents with disability are working separately to those without disability (for those attending mainstream schools). Students with special needs in Australian mainstream secondary schools typically attend three, 2-hour support lessons per week. Teachers will be asked to facilitate the delivery of at least two exercise sessions per week in Phases 1-3. Adapted from the original B2L intervention, teachers/participants will be able to select from pre-designed themed HIIT workouts that include a combination of foundational resistance exercises (i.e., push up, squat, front support, lunge) and aerobic exercises (i.e., shuttle run, high knees running on the spot). The sessions last 10 to 20 minutes including an appropriate warm-up. Due to the wide range of abilities expected from the participants, teachers will be encouraged to adapt the specific types of exercises and task complexity for each student. The task complexity and variations within the HIIT sessions will progressively increase over the study period. To monitor exercise intensity, students will be equipped with heart rate sensors (Polar Verity Sense) that will pair with a purpose-built iPad application (hereafter 'app') using Bluetooth connectivity. Students will be encouraged to reach a target intensity of ≥80% of age-predicted heart rate maximum during the work intervals. As demonstrated in our feasibility study, this heart rate target is achievable for students with disability [24].

For peer review only - http://bmjopen.bmj.com/site/about/guidelines.xhtml

Smartphone app: Teachers and students will be provided with access to a bespoke smartphone app available via both Android and iOS operating systems. The app includes: (i) a teacher version that allows whole-class heart rate monitoring during 'class' sessions, (ii) descriptions and depictions of exercise sessions, (iii) options for 'solo' or 'group' sessions, (iv) timer, audible prompts and display of heart rate using Bluetooth-synced heart rate monitors during HIIT sessions, and (v) personalised reports outlining heart rate. For students who do not own a smartphone, access to the B2L app will be provided to parents. During the professional development workshop, teachers will be encouraged to deliver school-based sessions using the teacher version that allows whole-class heart rate monitoring. Students will be encouraged to use the app during activity sessions outside of the school setting.

Parent support videos: Parents will receive two e-newsletters containing links to video overviews of B2La, the benefits and barriers of physical activity for individuals with disability, and strategies to support their children's participation in physical activity outside of school. The e-newsletters will be delivered to parents in Phases 1 and 2 of the intervention.

Intervention conceptual model and theoretical frameworks

B2La was guided by the conceptual model proposed by Lubans and colleagues [31], which includes four complementary tenets that are fundamental to the successful scale-up of adolescent HIIT interventions.

Opportunity: The B2La sessions will be delivered during scheduled 'Specialist Support Classes' (students typically attend 3 x 2-hour support lessons/week). These classes cater for students with moderate-to-high learning and support needs, including students with intellectual disability, mental health issues, autism, physical disability, sensory impairment, and behaviour disorders. Based on our formative research with teachers and the NSW Department of Education, 'Specialist Support Classes' represent an ideal 'new' opportunity [32] for the delivery of B2La.

Design: The school-based exercise sessions will provide participants with opportunities to collaboratively develop their exercise competence, confidence, and knowledge. Participants will also be provided with opportunities to design and run their own HIIT sessions. The study information seminar will focus on the benefits and barriers of physical activity for individuals with disability [4] as well as evidence-based behaviour change techniques.

For peer review only - http://bmjopen.bmj.com/site/about/guidelines.xhtml

Delivery: B2La has been guided by Self-Determination Theory (SDT) to enhance students' autonomous motivation for physical activity [33]. Aligned with SDT, teachers will be provided with training and support to deliver the B2La sessions using the 'Supportive, Active, Autonomous, Fair and Enjoyable' (SAAFE) principles [34]. Participants' need for autonomy will be satisfied by providing opportunities for choice within sessions (e.g., type of activity, music playing, and training partner). Competence will be satisfied using positive and skill-specific feedback from teachers, with a focus on effort over performance (via heart rate feedback). Schools will also be provided with resources designed to support the accessibility, engagement, and development of exercise skills (e.g., technique cards, B2L app). Teachers will utilise a variety of strategies to enhance group cohesion and satisfy students' needs for relatedness during HIIT sessions (i.e., encouraging supportive behaviour among students such as 'high fives' and facilitating partner work) [35].

Support: The implementation of B2La will be supported by the Consolidated Framework for Implementation Research (CFIR) [36]. Strategies used to facilitate the implementation of the B2La intervention will cover the five CFIR domains: intervention characteristics, outer setting (educational authorities), inner setting (schools), characteristics of individuals (teachers), and the implementation process (see Table 1).

Table 1: Strategies used to facilitate implementation in the Burn 2 Learn adapted intervention

Domains	Constructs	Strategies
**B2La intervention characteristics**	Evidence strength and quality	Findings from *B2L* cluster RCT and *B2La* feasibility study used in promotional and training materials.
	Adaptability	Flexible intervention delivery model (i.e., during class-time, breaks, before or after school) requiring minimal access to facilities (i.e., can be done in the classroom) and equipment (i.e., body weight exercises).
	Complexity	Time-efficient intervention requiring only two or three 15-20 minute sessions per week.
	Design quality and packaging	*B2La* program resources developed by a professional graphic designer. Multimedia designed using evidence-based principles for learning.
**Outer setting (Educational authorities)**	Partnerships and investment	Partnership with the NSW Department of Education and Special Olympics Australia.
	External policy and incentives	Professional learning accreditation with state-based educational standards authority.
**Inner setting (Schools)**	School culture	Teachers will be encouraged to give a presentation to school staff focused on the benefits of activity for students' mental health and academic outcomes.

For peer review only - http://bmjopen.bmj.com/site/about/guidelines.xhtml

	Leadership engagement	Teachers and external change agents will meet with the school principal to ensure commitment.
	Equipment	Schools will be provided with a basic equipment pack (~$2,000 AUD).
	Relative priority	Promoted to schools as strategy to improve cognitive function and mental health.
**Characteristics of individuals (Teachers)**	Self-efficacy, knowledge, and beliefs (teacher)	Full day professional development workshop provided for teachers. Online version of workshop available.
	Perceived barriers (students)	Designed to be time efficient, and motivating for students, through the SAAFE teaching principles.
**Implementation in process**	Planning for implementation	Teachers required to complete an action plan to support B2La implementation in their school.
	Champions	Recruitment of two school champions at each intervention school.
	External support agents	Schools will be allocated external change agent, who will visit twice for planning and evaluation.
	Evaluation and feedback	External change agents will conduct session observations and provide feedback to teachers.

Note. B2La = Burn 2 Learn *adapted*

Teachers recruited as school champions will attend a one-day professional learning workshop led by members of the research team. The workshop will provide teachers with the training and resources needed to facilitate school-based HIIT sessions. The workshop will involve a combination of theoretical (i.e., program rationale, benefits of HIIT, school implementation plan) and practical (e.g., participation in a B2La HIIT session, peer assessment of exercise technique, and overview of how to use program resources) activities.

Measures and data collection

Trained research assistants, blinded to group allocation at all time-points, will conduct assessments for the primary outcome. Questionnaires will be completed with the assistance of research assistants using electronic tablets. Physical assessments will be conducted in a sensitive manner by a research assistant of the same sex where possible. Standard demographic information (e.g., age, sex, ethnicity, country of birth, residential postcode, and parent/caregivers' education level) will be collected at baseline. All measurements will be conducted at baseline, 6-months post-baseline (primary endpoint) and 9-months post-baseline. The only exception will be students' on-task behaviour, which will be assessed at baseline and mid-intervention (3-months post-baseline), and cognitive function which will be assessed at baseline and 6-months only. Of note, due to physical and intellectual limitations,

For peer review only - http://bmjopen.bmj.com/site/about/guidelines.xhtml

not all participants will be able to complete all measures and modifications will be made, as necessary.

Primary outcome

Functional capacity. Consistent with previous physical activity interventions targeting youth with disability [37], our primary outcome is functional capacity, assessed using the 6MWT [38], which has good reliability in adolescents with disability (ICC =0.82) [27]. The 6-Minute Push Test will be used for students who self-propel a wheelchair [39]. Students will be instructed to cover as much distance as possible in 6 minutes and the distance (in metres) covered will be documented.

Secondary outcomes

Physical activity. Participants will be instructed to wear an ActiGraph GT9X Link accelerometer on their non-dominant wrist for 24 hours/day (even when bathing, swimming, and sleeping) for a period of seven consecutive days (3-day minimum wear time). School hour, weekday and weekend (i.e., mean minutes per day) physical activity will be calculated separately, using existing thresholds for categorising physical activity intensity [22].

Muscular fitness. Lower body muscular endurance will be assessed using the 30 second sit-to-stand test [40]. From a seated position, students will attempt to stand up and sit back down on a 44cm high bench seat as many times as possible in 30 seconds [18]. A modified version of the 90-degree push-up test will be used to assess upper body muscular endurance [41]. All students will be instructed to perform as many push-ups as possible on their knees.

Body composition. Body weight and height will be measured using a portable digital scale (A&D Medical UC-352-BLE Digital Scales) and a portable stadiometer (Seca 213 Portable Height Measuring Rod Stadiometer), respectively. Body mass index (BMI) will be calculated using the standard formula (weight[kg]/height[m]^2). Age- and sex-specific BMI z-scores will be calculated, and participants will be classified into weight categories according to International Obesity Task Force cut-offs [42].

For peer review only - http://bmjopen.bmj.com/site/about/guidelines.xhtml

Cognitive function. This will be assessed with electronic tablets using the cognitive portion of the National Institutes of Health (NIH) Toolbox [43]. The Toolbox has been used with children and adults with Fragile X syndrome, Down syndrome, and intellectual disabilities, with tests demonstrating good to excellent reliability and feasibility [43, 44]. Participants will complete the Flanker (inhibition), list sorting (working memory) and dimensional change card sort (cognitive flexibility) tasks.

Quality of life. Health-related quality of life will be assessed using the Child Health Utility 9-Dimensions [45], which includes 9-items (worried, sad, pain, tired, annoyed, schoolwork or work, sleep, daily routine, and activities) each item scored on a 5-point scale.

Physical literacy. Autonomous motivation for physical activity will be assessed using identified and intrinsic subscales from the 'Behavioural Regulations in Exercise Questionnaire-2 [46]. Confidence will be assessed using the validated 6-item High-Intensity Interval Training Self-efficacy Questionnaire [47]. Competence will be assessed using video analysis of a selection of skills from the Resistance Training Skills Battery (i.e., push-ups, lunge, squat, and front support chest touch), which has been validated among typically developing adolescents [48] and among children with varying degrees of motor skill proficiency [49].

Externalising behaviours. Teachers will complete a Student Behaviour Questionnaire [50] for each student at baseline, 6- and 9-months. The questionnaire consists of ten statements, regarding students' classroom behaviours, observed over the previous 6-months, which are rated using a 3-point Likert scale. The items have been adapted from the Strengths and Difficulties questionnaire externalising subscale.

On-task behaviour. To determine the acute effect of the B2La intervention on students' behaviour in the classroom, observations will be conducted by trained research assistants at baseline and mid-intervention (3-months) using established methods [51]. The assessment includes a 30-minute observation period where research assistants will assess the on- and off-task behaviour of six randomly selected students (5 min per student). Observation and recording are completed in 15-sec intervals (20 observations per student) and teachers and students will not know who is being observed during the assessment.

For peer review only

Economic evaluation

We will assess the efficiency and affordability of the intervention using cost-effectiveness/cost utility analysis and budget impact respectively, conducted from a public finance perspective. The effectiveness measure will be based on the primary outcome (6MWT). Transformation of the CHUI-9D data will be employed in a cost utility analysis. The resource use and costs for the intervention and usual practice will be prospectively measured and derived from project records (staff and consumables), teacher surveys and school records. Additional costs in the intervention group are anticipated to be labour (implementation support), program development, and training costs. The cost effectiveness analysis will be conducted on a 'within trial' basis, that is, over the 6-month study period, comparing incremental costs and outcomes. Affordability of the program will be calculated using budget impact analysis, over a standard accounting cycle and is designed to assist decision making in schools and hence assist the translation of cost-effective and affordable programs. Scenario analysis will assess the costs to implement the program at scale across NSW. Reporting for the economic analysis will adhere to the CHEERS guidelines [52].

Process evaluation

We will conduct a process evaluation to determine program acceptability, implementation, adaptability, and sustainability in schools.

Acceptability: We will conduct focus groups to determine teachers' and participants' (i.e., students) perceptions of, and experiences with, the intervention. Teachers will also complete the Acceptability of Intervention Measure, Intervention Appropriateness Measure, and Feasibility of Intervention Measure [53].

Implementation: Teachers will be asked to record their delivery of B2La sessions using the teacher handbook. We will also track the number of sessions delivered using the B2La smartphone app. Members of our research team will conduct two session observations (using the SAAFE checklist) at each school to determine intervention fidelity. Finally, participants' mean heart rate during sessions will collected using the B2L app.

Adaptability: Teachers will be asked to reflect on how they adapted the intervention in the focus groups. This will include adaptions in relation to the characteristics of the school, class, and students.

For peer review only - http://bmjopen.bmj.com/site/about/guidelines.xhtml

Sustainability: Sustainability will be explored in the focus groups and via teacher and participant post-program evaluation questionnaires. Teachers will report their intention to deliver B2L in the future and complete an adapted version of the Program Sustainability Assessment Tool [54, 55]. Students will report their intention participate in HIIT in the two months following program completion.

Statistical analyses

Blinded analyses of the primary and secondary outcomes will be conducted by an independent statistician, using linear mixed models SAS V 9.1 (SAS Institute Inc, Cary, NC), with alpha levels set at $p<0.05$. The models will be used to assess the impact of group (B2La or control), time (treated as categorical with levels baseline, 6- and 9-months), and the group-by-time interaction. The models will include a random intercept for participant to account for the repeated measures for each participant, and a random intercept for school to account for the clustered design. The primary endpoint of the study will be 6-months from baseline. Least square mean differences between the treatment groups will be presented at both follow-up time points, with 95% confidence intervals and *p*-values. Compared to complete case analyses, mixed models include available data for all participants and are thus both more efficient and robust to bias. Mixed model analyses are consistent with the intention-to-treat principle, assuming the data are missing at random. The validity of this assumption will be explored by assessing relationships between missingness and observed values. We will conduct two sensitivity analyses for the primary outcome: (i) multiple imputation (assuming data are missing at random) and (ii) complete-case analysis (assuming data are missing completely at random). Four potential moderators (i.e., SES, sex, initial weight status and disability type) will be explored using interaction terms (i.e., time-by-treatment-by-moderator). If an interaction term is significant ($p<0.1$), sub-group analyses will be conducted.

Data monitoring

An internal monitoring committee consisting of DRL (Lead), AAL, and the Project Manager, will oversee the conduct of the study and manage any data or safety issues that may arise. All entered data will be de-identified using participant codes and will be stored electronically in a password protected drive at the University of Newcastle. Data will be checked for implausible values and 20% of the data will be entered twice to confirm accuracy. It is not

For peer review only - http://bmjopen.bmj.com/site/about/guidelines.xhtml

expected that participants will be at any greater risk of adverse events than they would be when participating in other types of school-based physical activity. However, the teacher handbook includes a section for teachers to report any injuries or adverse events that may occur. Any adverse events will be documented and reported to the relevant ethics committee. Any amendments to the study protocols will be publicly available via the Australian and New Zealand Clinical Trials Registry (Trial number: ACTRN12621000884808). We have not included any formal guidelines for stopping the trial early, as we have not planned a formal interim analysis of the primary outcome.

DISCUSSION

Burn 2 Learn adapted has been designed to provide older adolescents with disability an opportunity to be active at school, but also focuses on developing their physical literacy (i.e., physical competence, confidence, knowledge, and motivation) to engage in vigorous physical activity. Importantly, our research team will provide teachers with training and support to ensure that the program is delivered in an engaging manner that supports students' autonomous motivation to be active across the lifespan. Most HIIT studies have been delivered by researchers in controlled settings to establish efficacy, with little consideration of how they will work in the 'real world' [31]. By comparison, B2La was designed with scale-up in mind using the Consolidated Framework for Implementation Research to support implementation and sustainability. This may help to reduce the 'voltage drop' that typically occurs as interventions progress from efficacy to effectiveness to dissemination [29, 56-58].

The strengths of our study include the cluster randomised controlled trial design. We also consider our intervention design to be a strength, as it was developed in consultation with adolescents with disability and key stakeholders (i.e., NSW Department of Education and Special Olympics Australia). Our comprehensive assessment of physical, mental, and cognitive health is an additional study strength. However, there are potential limitations that should be noted. First, COVID-19 is still a major problem in Australian schools, resulting in high levels of teacher and student absenteeism. This is likely to affect recruitment, data collection, and intervention implementation. Second, having a unique study population with physical and/or intellectual limitations, not all participants will be able to complete all measures. Finally, it might not be possible to retain the blinding of all research assistants at post-test assessments.

For peer review only - http://bmjopen.bmj.com/site/about/guidelines.xhtml

ETHICS AND DISSEMINATION

Ethics approval for this cluster RCT was obtained from the Human Research Ethics Committee of the University of Newcastle, Australia (H-2021-0262) and the NSW Department of Education and Communities (SERAP:2021257). School Principals, teachers, parents, and students will all provide informed written consent prior to enrolment. Example participant information and consent forms are provided in our supplementary materials. The full protocol, participant-level dataset, and statistical code will be available upon request from DRL. We will publish our findings in peer-reviewed journals and provide the NSW Department of Education and all participating schools with a detailed report at the conclusion of the trial. If the intervention is successful, we will support dissemination via a series of professional learning workshops for teachers in NSW schools.

Contributors: TK: investigation, resources, data curation, writing-original draft. AAL: methodology, investigation, resources, data curation, writing-review and editing, funding acquisition. JJS: methodology, investigation, resources, writing-review and editing, funding acquisition. NE: methodology, investigation, resources, writing-review and editing, funding acquisition. NS: methodology, resources, writing-review and editing, funding acquisition. MN: methodology, resources, writing-review and editing, funding acquisition. CL: methodology, writing-review and editing, funding acquisition. CHH: methodology, writing-review and editing, funding acquisition. PR: writing-review and editing, guided statistical analysis, funding acquisition. CO: writing-review and editing, guided statistical analysis. SGK: resources, writing-review and editing. JB: resources, writing-review and editing. LS: resources, writing-review and editing. PC: resources, writing-review and editing. LR: resources, data curation, writing-review and editing. DRL: conceptualisation, methodology, investigation, resources, writing-original draft, supervision, funding acquisition.

Competing interests: None declared.

Funding: The study is funded by Medical Research Future Fund (APP2007095). DRL is funded by a National Health and Medical Research Council Senior Research Fellowship (APP1154507). This project is supported and co-designed with the NSW Department of Education and Special Olympics Australia. The study funders will have no role in data

For peer review only - http://bmjopen.bmj.com/site/about/guidelines.xhtml

collection, analysis, interpretation, or writing. Nor will they influence over the publication of findings.

For peer review only

For peer review only - http://bmjopen.bmj.com/site/about/guidelines.xhtml

REFERENCES

1. World Health Organisation, *Towards a Common Language for Functioning, Disability and Health*. 2002, World Health Organization: Geneva.
2. World Health Organization, *World report on disability 2011*. 2011, World Health Organization: Geneva.
3. World Health Organization, *WHO global disability action plan 2014-2021: better health for all people with disability*. 2015, World Health Organization: Geneva.
4. Hassett, L., et al., *Comparisons of leisure-Time physical activity participation by adults with and without a disability: Results of an Australian cross-sectional national survey.* BMJ Open SEM, 2021. **7**: p. e000991.
5. McVilly, K., et al., *Diabetes in people with an intellectual disability: a systematic review of prevalence, incidence and impact.* Diabet Med, 2014. **31**(8): p. 897-904.
6. Rimmer, J.H., et al., *Obesity and obesity-related secondary conditions in adolescents with intellectual/developmental disabilities.* J Intellect Disabil Res, 2010. **54**(9): p. 787-94.
7. McKenzie, G., C. Willis, and N. Shields, *Barriers and facilitators of physical activity participation for young people and adults with childhood-onset physical disability: a mixed methods systematic review.* Dev Med Child Neurol, 2021. **63**(8): p. 914-924.
8. Shields, N. and A. Synnot, *Perceived barriers and facilitators to participation in physical activity for children with disability: a qualitative study.* BMC Pediatrics, 2016. **16**(1): p. 9.
9. Wright, A., et al., *Barriers and facilitators to physical activity participation for children with physical disability: comparing and contrasting the views of children, young people, and their clinicians.* Disabil Rehabil, 2019. **41**(13): p. 1499-1507.

For peer review only - http://bmjopen.bmj.com/site/about/guidelines.xhtml

10. McGarty, A.M., et al., *A systematic review and meta-analysis of interventions to increase physical activity in children and adolescents with intellectual disabilities.* J Intellect Disabil Res, 2018. **62**(4): p. 312-329.

11. Hills, A.P., D.R. Dengel, and D.R. Lubans, *Supporting public health priorities: recommendations for physical education and physical activity promotion in schools.* Prog Cardiovasc Dis, 2015. **57**(4): p. 368-74.

12. Rekaa, H., H. Hanisch, and B. Ytterhus, *Inclusion in Physical Education: Teacher Attitudes and Student Experiences. A Systematic Review.* Intl J Disabil Dev Educ, 2019. **66**(1): p. 36-55.

13. Haegele, J. and S. Sutherland, *Perspectives of Students with Disabilities Toward Physical Education: A Qualitative Inquiry Review.* Quest -Illinois- National Association for Physical Education in Higher Education-, 2015. **67**: p. 255-273.

14. Hardman, K., et al., *World-wide survey of school physical education.* 2013.

15. Naylor, P.J., et al., *Implementation of school based physical activity interventions: a systematic review.* Prev Med, 2015. **72**: p. 95-115.

16. Costigan, S., et al., *High-intensity interval training for improving health-related fitness in adolescents: A systematic review and meta-analysis.* BJSM, 2015. **49**.

17. Leahy, A., et al., *Review of High-Intensity Interval Training for Cognitive and Mental Health in Youth.* MSSE, 2020. **52**: p. 1.

18. Boer, P.H., et al., *The influence of sprint interval training on body composition, physical and metabolic fitness in adolescents and young adults with intellectual disability: a randomized controlled trial.* Clin Rehabil, 2014. **28**(3): p. 221-31.

19. Zwinkels, M., et al., *Effects of High-Intensity Interval Training on Fitness and Health in Youth With Physical Disabilities.* Pediatr Phys Ther, 2018. **31**: p. 1.

For peer review only - http://bmjopen.bmj.com/site/about/guidelines.xhtml

20. Leahy, A.A., et al., *School-based physical activity intervention for older adolescents: rationale and study protocol for the Burn 2 Learn cluster randomised controlled trial.* BMJ Open, 2019. **9**(5): p. e026029.

21. Leahy, A.A., et al., *Feasibility and Preliminary Efficacy of a Teacher-Facilitated High-Intensity Interval Training Intervention for Older Adolescents.* Pediatr Exerc Sci, 2019. **31**(1): p. 107-117.

22. Lubans, D., et al., *Time-efficient intervention to improve older adolescents' cardiorespiratory fitness: findings from the 'Burn 2 Learn' cluster randomised controlled trial.* BJSM, 2020. **55**: p. bjsports-2020.

23. Mavilidi, M.F., et al., *Effect of a Time-Efficient Physical Activity Intervention on Senior School Students' On-Task Behaviour and Subjective Vitality: the 'Burn 2 Learn' Cluster Randomised Controlled Trial.* Educ Psychol Rev, 2021. **33**(1): p. 299-323.

24. Leahy, A., et al., *Feasibility of a school-based physical activity intervention for adolescents with disability.* Pilot and Feasibility Studies, 2021. **7**.

25. Moher, D., et al., *CONSORT 2010 Explanation and Elaboration: updated guidelines for reporting parallel group randomised trials.* BMJ, 2010. **340**: p. c869.

26. McConkey, R. and J. Walsh, *An index of social competence for use in determining the service needs of mentally handicapped adults.* J Ment Defic Res, 1982. **26**(Pt 1): p. 47-61.

27. Elmahgoub, S.S., et al., *Reproducibility, validity and predictors of six-minute walk test in overweight and obese adolescents with intellectual disability.* Disabil Rehabil, 2012. **34**(10): p. 846-51.

For peer review only - http://bmjopen.bmj.com/site/about/guidelines.xhtml

28. Bartels, B., J.F. de Groot, and C.B. Terwee, *The six-minute walk test in chronic pediatric conditions: a systematic review of measurement properties.* Phys Ther, 2013. **93**(4): p. 529-41.

29. Beets, M.W., et al., *Identification and evaluation of risk of generalizability biases in pilot versus efficacy/effectiveness trials: a systematic review and meta-analysis.* IJBNPA, 2020. **17**(1): p. 19.

30. Australian Curriculum Assessment and Reporting Authority, *Guide to understanding the Index of Community Socioeducational Advantage (ICSEA)*, My School, Editor. 2020.

31. Lubans, D.R., et al., *Scaling-up Adolescent High-Intensity Interval Training Programs for Population Health.* Exerc Sport Sci Rev, 2022. 50(3), 128-136.

32. Beets, M.W., et al., *The theory of expanded, extended, and enhanced opportunities for youth physical activity promotion.* Int J Behav Nutr Phys Act, 2016. **13**(1): p. 120.

33. Ryan, R.M. and E.L. Deci, *Self-determination theory: Basic psychological needs in motivation, development, and wellness*. Self-determination theory: Basic psychological needs in motivation, development, and wellness. 2017, New York, NY, US: The Guilford Press. xii, 756-xii, 756.

34. Lubans, D.R., et al., *Framework for the design and delivery of organized physical activity sessions for children and adolescents: rationale and description of the 'SAAFE' teaching principles.* Int J Behav Nutr Phys Act, 2017. **14**(1): p. 24.

35. K, O.B., et al., *Self-determined motivation and physical activity in children and adolescents: a systematic review and meta-analysis.* Prev Med, 2014. **67**: p. 270-9.

36. Damschroder, L.J., et al., *Fostering implementation of health services research findings into practice: a consolidated framework for advancing implementation science.* Implement Sci, 2009. **4**: p. 50.

For peer review only - http://bmjopen.bmj.com/site/about/guidelines.xhtml

37. Shields, N., et al., *FitSkills: protocol for a stepped wedge cluster randomised trial of a community-based exercise programme to increase participation among young people with disability.* BMJ Open, 2020. **10**(7): p. e037153.

38. Wouters, M., H.M. Evenhuis, and T.I. Hilgenkamp, *Systematic review of field-based physical fitness tests for children and adolescents with intellectual disabilities.* Res Dev Disabil, 2017. **61**: p. 77-94.

39. Damen, K.M.S., et al., *6-Minute Push Test in Youth Who Have Spina Bifida and Who Self-Propel a Wheelchair: Reliability and Physiologic Response.* Phys Ther, 2020. **100**(10): p. 1852-1861.

40. Boer, P.H. and S.J. Moss, *Test-retest reliability and minimal detectable change scores of twelve functional fitness tests in adults with Down syndrome.* Res Dev Disabil, 2016. **48**: p. 176-85.

41. Winnick, J.P. and F.X. Short, *Brockport Physical Fitness Test Manual : A Health-Related Assessment for Youngsters With Disabilities*, in *Brockport Physical Fitness Test Manual : A Health-Related Assessment for Youngsters With Disabilities*, J.P. Winnick and F.X. Short, Editors. 2014, Human Kinetics: Champaign, IL.

42. Cole, T.J. and T. Lobstein, *Extended international (IOTF) body mass index cut-offs for thinness, overweight and obesity.* Pediatr Obes, 2012. **7**(4): p. 284-94.

43. Weintraub, S., et al., *Cognition assessment using the NIH Toolbox.* Neurology, 2013. **80**(11 Suppl 3): p. S54-64.

44. Hessl, D., et al., *The NIH Toolbox Cognitive Battery for intellectual disabilities: three preliminary studies and future directions.* J Neurodev Disord, 2016. **8**(1): p. 35.

45. Stevens, K. and J. Ratcliffe, *Measuring and valuing health benefits for economic evaluation in adolescence: an assessment of the practicality and validity of the child*

For peer review only - http://bmjopen.bmj.com/site/about/guidelines.xhtml

health utility 9D in the Australian adolescent population. Value Health, 2012. **15**(8): p. 1092-9.

46. Markland, D. and V. Tobin, *A Modification to the Behavioural Regulation in Exercise Questionnaire to Include an Assessment of Amotivation.* JSEP, 2004. **26**(2): p. 191-196.

47. Eather, N., et al., *Development and Evaluation of the High-Intensity Interval Training Self-Efficacy Questionnaire.* J Sport Exerc Psychol, 2020: p. 1-9.

48. Lubans, D.R., et al., *Development, test-retest reliability, and construct validity of the resistance training skills battery.* J Strength Cond Res, 2014. **28**(5): p. 1373-80.

49. Bebich-Philip, M.D., et al., *Adaptation of the Resistance Training Skills Battery for Use in Children Across the Motor Proficiency Spectrum.* Pediatr Exerc Sci, 2016. **28**(3): p. 473-80.

50. Mellor, D., *Normative data for the Strengths and Difficulties Questionnaire in Australia.* Aust Psychol, 2005. **40**: p. 215-222.

51. Alerto, P.A. and A.C. Troutman, *Applied Behavior Analysis for Teachers*. 2003, Pearson: Australia.

52. Husereau, D., et al., *Consolidated Health Economic Evaluation Reporting Standards 2022 (CHEERS 2022) statement: updated reporting guidance for health economic evaluations.* BJOG, 2022. **129**(3): p. 336-344.

53. Weiner, B.J., et al., *Psychometric assessment of three newly developed implementation outcome measures.* Implement Sci, 2017. **12**(1): p. 108.

54. Hall, A., et al., *Adaptation and Validation of the Program Sustainability Assessment Tool (PSAT) for Use in the Elementary School Setting.* Int J Environ Res Public Health, 2021. **18**(21).

For peer review only

For peer review only - http://bmjopen.bmj.com/site/about/guidelines.xhtml

55. Luke, D., et al., *The Program Sustainability Assessment Tool: A New Instrument for Public Health Programs.* Prev. Chronic Dis., 2014. **11**: p. E12.

56. Chambers, D., R. Glasgow, and K. Stange, *The dynamic sustainability framework: Addressing the paradox of sustainment amid ongoing change.* Implementation science : IS, 2013. **8**: p. 117.

57. Lane, C., et al., *How effective are physical activity interventions when they are scaled-up: a systematic review.* Int J Behav Nutr Phys Act, 2021. **18**(1): p. 16.

58. McCrabb, S., et al., *Scaling-up evidence-based obesity interventions: A systematic review assessing intervention adaptations and effectiveness and quantifying the scale-up penalty.* Obes Rev, 2019. **20**(7): p. 964-982.

For peer review only

For peer review only - http://bmjopen.bmj.com/site/about/guidelines.xhtml

Figure Legend

Figure 1. Example of B2La HIIT technique card

This figure was created by the lead investigator

Figure 2. Example of B2La HIIT technique card

This figure was created by the lead investigator

For peer review only

For peer review only - http://bmjopen.bmj.com/site/about/guidelines.xhtml

Figure 1: Example B2La HIIT technique card

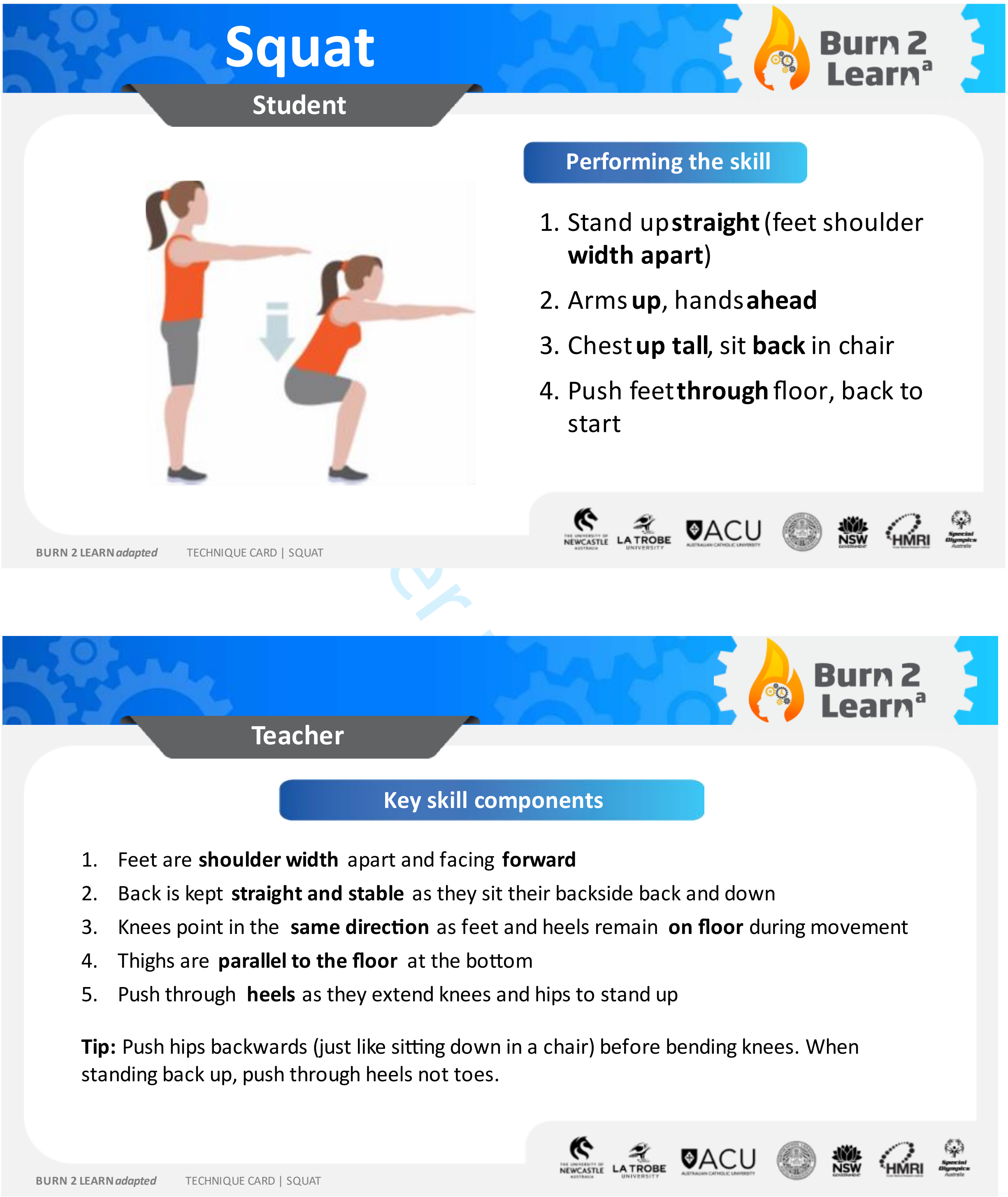

Figure 2: Example B2La HIIT task cards

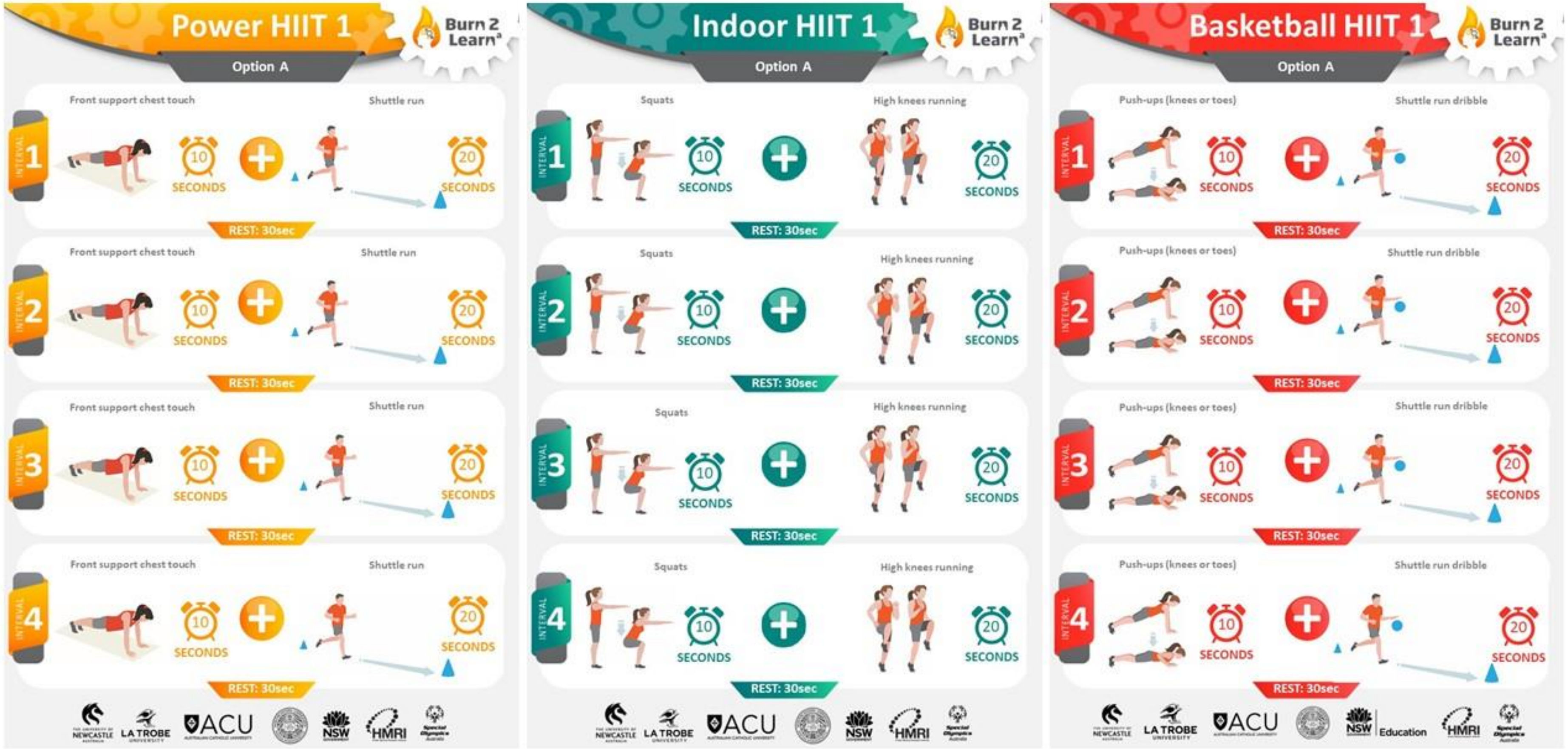

For peer review only

Professor David Lubans
School of Education
Faculty of Education and Arts
University of Newcastle
Callaghan NSW 2308
Phone: + 61 (0)2 4921 2049
Fax: +61 (0)2 4921 7407
Email: david.lubans@newcastle.edu.au

THE UNIVERSITY OF NEWCASTLE AUSTRALIA

Research Project: Increasing physical activity and improving fitness to support the wellbeing of senior school students

Document Version 7; dated 13/05/2020

STUDENT & PARENT INFORMATION STATEMENT

Dear student and parent/guardian,

You are invited to participate in the research project identified above which is being conducted by Prof David Lubans and colleagues from the University of Newcastle and Australian Catholic University. This research study is being funded by a National Health and Medical Research Council (NHMRC) project grant.

Why is this research being done?

Physical activity declines sharply during adolescence and findings reported in the 2016 Active Healthy Kids Australia (AHKA) Physical Activity Report Card suggest that only 18% of 12-17 year old youth meet national physical activity guidelines. The senior school years can be a time of significant stress for students, and recent national surveys show that a substantial proportion of year 11 and 12 students experience moderate to high levels of psychological distress. Regular physical activity has been shown to support young peoples' mental health, and there is growing evidence linking physical activity and fitness to cognitive functioning and academic performance. Despite these benefits, schools usually do not provide regular physical activity opportunities to students during the senior school years.

The primary aim of the study is to assess the impact of a physical activity program delivered in the school setting on students' fitness, well-being and cognitive functioning.

Who can participate in this research?

Grade 11 and 12 students from the Special Education Faculty, Callaghan College Senior Campus. We aim to recruit 20-25 students.

What choice do you have?

The program components will be delivered by teachers as part of regular school activity. This invitation relates to the evaluation of that program. Participation in the evaluation component of this study is entirely up to you and your parent(s)/guardian(s), and you can choose to participate in all, some or none of the evaluation measures. If you agree to participate you can choose to withdraw at any time. A decision not to participate or discontinuation of involvement in the study will not jeopardise your relationship with the University of Newcastle, Australian Catholic University or the school. Withdrawal from the evaluation component will not result in any disciplinary action, nor will it affect your academic grades, given that this is a purely voluntary research task.

What is involved in this study?

The program will run for 8-weeks and has been designed to improve students' physical and mental health. Program will start in Term 2 (week 6) and run into Term 3 (week 4). Consenting students will complete evaluation measures at baseline and 8-weeks. Students who do not consent to participate will not be involved in the evaluation component of the study. However, it is the school's decision to deliver the physical activity program as part of the school curriculum. Therefore, a decision not to participate in the program activities must be discussed with and decided upon by the school. The program components and evaluation measures are listed below in Table 1.

For peer review only

Table 1: Program components and evaluation strategies

Program components	***Evaluation of program***
**i) Interactive seminar:** Students will attend 1 interactive seminar delivered by the school champion, but supported by a member of the research team. The interactive seminar will provide an overview of Burn 2 Learn and will address relevant information regarding physical activity, mental health and cognition.<br><br>**ii) School-based exercise sessions:** Sessions will be run at school during class time. Schools will be encouraged to offer 3 sessions/week for 2 school terms (i.e., the colder months), and each session will last approximately 10-15 minutes in duration. The sessions will involve a combination of aerobic (e.g., shuttle runs, jumping jacks, boxing, dancing) and muscle strengthening exercises (e.g., push-ups, squat jumps and walking lunges), and have been designed to be fun and engaging as well as vigorous in nature.<br><br>**iii) Parental e-newsletter:** Parents will receive two e-newsletters containing information on the benefits of physical activity for academic performance and mental health and strategies to support their children's participation in physical activity during school holiday periods.<br><br>**iv) Smartphone app:** A smartphone app has been developed to enable students to complete the sessions at school and home. Android and iOS versions of the app will be available. The app includes: (i) descriptions of exercise sessions with timer, audible prompts and recording of heart rate results, (ii) ability to review session heart rate records, and (iii) self-monitoring/goal setting to promote participation in all types of physical activity.	The following measures will be taken twice (baseline and 8-weeks):<br><br>• *Health-related fitness:* Aerobic fitness will be tested using the 3 minute step test and muscular fitness will be assessed using the timed push up and sit to stand tests. Body composition will be assessed sensitively using height and weight (i.e., to calculate Body Mass Index [BMI]). Measurements will take place out of the view of other students, and students will have the choice of being assessed by either a male or female researcher.<br><br>• *Mental health:* will be measured using a questionnaire.<br><br>• *Sleep behaviour*: will be assessed using existing questionnaires.<br><br>• *Cognitive functioning:* will be assessed using computer-based tests that assess aspects of cognitive control.

How long will it take?

**Task**	**Approximate length of time**
Baseline and post-intervention questionnaires and testing	50-60 minutes at each time point
Physical activity sessions	Approximately 15 minutes/session, 3 times per week for 8-weeks.

What are the risks and benefits of participating?

The evaluation measures will be carried out by trained research assistants, and will be conducted in a sensitive manner at all times. The school-based exercise sessions will be developed by the research team and delivered by students. Based on previous studies, students will have no greater chance of injury by participating in these programs in comparison to other sports and physical activities. In the event of an injury occurring, the student will immediately be asked to stop participation, and normal school procedures for the management of injury will be followed. The student will not return to participation in the program's physical activities until clearance has been received from a suitable practitioner. If you have a health or medical condition precluding your participation in vigorous exercise, you SHOULD NOT participate in this program. If you are unsure if this applies to you, you and your parent/guardian should seek advice from a qualified medical professional (e.g., a General Practitioner) prior to consenting to participate. The program will provide students with an opportunity to increase their knowledge and skills and improve attitudes toward physical activity. Students will also benefit from participation in a variety of enjoyable exercise activities as part of the program's delivery. Questionnaires utilised during the program evaluation involve the use of a Psychological Distress Scale and other questions that individuals may find distressing. To manage this risk, students are advised on each survey that: "*Completing the questionnaires is entirely the choice of the participants. Some of the questions are of a personal nature. If you feel uncomfortable with any question you may leave it and*

move to the next question, or discontinue the survey," and are provided with the contact details for Lifeline. If your involvement in completing any of the surveys or activities in the program make you feel uncomfortable or cause you distress then please contact Lifeline on 13 11 14 and/or your School Counsellor. You can also contact the researchers (contact details at the end of this letter) to discuss your concerns confidentially.

How will the information collected be used?

The data collected from this study will be used for journal publications and conference presentations and to inform future practice for the design of valuable, evidence-based physical activity programs in schools.

How will privacy be protected?

Any personal information provided by students and parents will be confidential to the researchers. The results of the study will be published in general terms and will not allow the identification of individual students or schools. Once the data has been collected, de-identified using participant codes and entered into an electronic data file, questionnaires and other data collection sheets will be destroyed. Data will be stored for a minimum of 5 years on password protected files (only accessible to researchers).

What do you need to do to participate?

Take some time to read through this information statement and discuss your participation with a parent/guardian. All students wanting to participate in the evaluation component of this study will be required to return the accompanying consent form, signed by the student and a parent/guardian. If you consent to participate, please return the signed form as soon as possible to the school's front office, the Head Teacher of the PE faculty, or your Year Advisor. If you are 18 years or older, you will not need a parent/guardian to provide consent. However, we still encourage you to discuss your involvement with a trusted adult prior to agreeing to participate.

Further information

Following the completion of the study, the school will be sent a report describing the findings of the study. Participants can request/access a summary of the results of the research by contacting Prof. David Lubans via phone or email (contact details below). Individual results will not be given to students. If you would like further information please do not hesitate to contact Prof David Lubans by email (david.lubans@newcastle.edu.au) or phone (02 4921 2049). Thank you for considering this invitation.

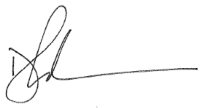

Prof David Lubans

Should you have concerns about your rights as a participant in this research, or you have a complaint about the manner in which the research is conducted, it may be given to the researcher, or, if an independent person is preferred, to the Human Research Ethics Officer, Research & Innovation Services, The University of Newcastle, University Drive, Callaghan NSW 2308, Australia, telephone (02) 49216333, email Human-Ethics@newcastle.edu.au.

This project has been approved by the University of Newcastle's Human Research Ethics committee, Approval number [H-2016-0424].

For peer review only

Professor David Lubans
School of Education
Faculty of Education and Arts
University of Newcastle
Callaghan NSW 2308
Phone: + 61 (0)2 4921 2049
Fax: +61 (0)2 4921 7407
Email: David.Lubans@newcastle.edu.au

THE UNIVERSITY OF NEWCASTLE AUSTRALIA

Research Project: Increasing physical activity and improving fitness to support the wellbeing of senior school students
STUDENT / PARENT CONSENT FORM

Chief Investigators: Prof David Lubans, Prof Philip Morgan, Prof Ronald Plotnikoff, Prof Chris Lonsdale, Prof Michael Nilsson, Dr Jordan Smith, Dr Narelle Eather

I have been given information about the project identified above and have discussed it with the student. We understand that if I consent to the student's involvement, he/she will participate in the study entitled: *Increasing physical activity and improving fitness to support the wellbeing of senior school students*.

We understand that the student will complete the following program evaluation measures: weight, height, cardio-respiratory fitness, muscular fitness, mental health, sleep behaviour, and cognitive functioning.

We have had an opportunity to ask Prof Lubans questions about the research. I have discussed this project with the student and we understand that their participation in this research is voluntary and that he/she is free to withdraw from all or part of the evaluation component at any time. His/her refusal to participate or withdrawal of consent will not affect his/her relationship with the University of Newcastle, Australian Catholic University or the school. A decision to withdraw will not result in any disciplinary action against the student, nor will it affect his/her academic grades, given that this is a purely voluntary research task.

Please discuss the project together and ensure that you are both happy for the student to participate before signing the consent form. By signing below I am indicating consent / assent to participate in this research project conducted by Prof David Lubans, as it has been described to us in the Information Statement, a copy of which I have retained.

Student name: ____

Student's signature: ______________________________ **Date:** _____________________

Student's mobile number (if applicable): _______________________
(this will be used to send a reminder regarding program session days/times).

Parent/guardian name: ______________________________________

Parent Phone: ____

Parent's Signature: _____________________________ **Date:** _____________________

Please sign the completed consent letter and return to the school office or Special Education Faculty.

Should you have concerns about your rights as a participant in this research, or you have a complaint about the manner in which the research is conducted, it may be given to the researcher, or, if an independent person is preferred, to the Human Research Ethics Officer, Research & Innovation Services, The University of Newcastle, University Drive, Callaghan NSW 2308, Australia, telephone (02) 49216333, email Human-Ethics@newcastle.edu.au.

This project has been approved by the University of Newcastle's Human Research Ethics Committee, Approval number [H-2016-0424].

For peer review only

STANDARD PROTOCOL ITEMS: RECOMMENDATIONS FOR INTERVENTIONAL TRIALS

SPIRIT 2013 Checklist: Recommended items to address in a clinical trial protocol and related documents*

Section/item	Item No	Description	Addressed on page number
**Administrative information**			
Title	1	Descriptive title identifying the study design, population, interventions, and, if applicable, trial acronym	_1___________
Trial registration	2a	Trial identifier and registry name. If not yet registered, name of intended registry	_3___________
	2b	All items from the World Health Organization Trial Registration Data Set	__Included in trial registration
Protocol version	3	Date and version identifier	__7___________
Funding	4	Sources and types of financial, material, and other support	_18__________
Roles and responsibilities	5a	Names, affiliations, and roles of protocol contributors	_1___________
	5b	Name and contact information for the trial sponsor	_18___________
	5c	Role of study sponsor and funders, if any, in study design; collection, management, analysis, and interpretation of data; writing of the report; and the decision to submit the report for publication, including whether they will have ultimate authority over any of these activities	_____18_________
	5d	Composition, roles, and responsibilities of the coordinating centre, steering committee, endpoint adjudication committee, data management team, and other individuals or groups overseeing the trial, if applicable (see Item 21a for data monitoring committee)	__19-20_______

For peer review only

Introduction

Background and rationale	6a	Description of research question and justification for undertaking the trial, including summary of relevant studies (published and unpublished) examining benefits and harms for each intervention	_5-6_________
	6b	Explanation for choice of comparators	____9_________
Objectives	7	Specific objectives or hypotheses	_7___________
Trial design	8	Description of trial design including type of trial (eg, parallel group, crossover, factorial, single group), allocation ratio, and framework (eg, superiority, equivalence, noninferiority, exploratory)	_7___________

Methods: Participants, interventions, and outcomes

Study setting	9	Description of study settings (eg, community clinic, academic hospital) and list of countries where data will be collected. Reference to where list of study sites can be obtained	_8___________
Eligibility criteria	10	Inclusion and exclusion criteria for participants. If applicable, eligibility criteria for study centres and individuals who will perform the interventions (eg, surgeons, psychotherapists)	_8___________
Interventions	11a	Interventions for each group with sufficient detail to allow replication, including how and when they will be administered	_10-12_______
	11b	Criteria for discontinuing or modifying allocated interventions for a given trial participant (eg, drug dose change in response to harms, participant request, or improving/worsening disease)	__19-20________
	11c	Strategies to improve adherence to intervention protocols, and any procedures for monitoring adherence (eg, drug tablet return, laboratory tests)	______13_______
	11d	Relevant concomitant care and interventions that are permitted or prohibited during the trial	______NA______
Outcomes	12	Primary, secondary, and other outcomes, including the specific measurement variable (eg, systolic blood pressure), analysis metric (eg, change from baseline, final value, time to event), method of aggregation (eg, median, proportion), and time point for each outcome. Explanation of the clinical relevance of chosen efficacy and harm outcomes is strongly recommended	_14-15_______
Participant timeline	13	Time schedule of enrolment, interventions (including any run-ins and washouts), assessments, and visits for participants. A schematic diagram is highly recommended (see Figure)	_7___________

For peer review only - http://bmjopen.bmj.com/site/about/guidelines.xhtml

Sample size	14	Estimated number of participants needed to achieve study objectives and how it was determined, including clinical and statistical assumptions supporting any sample size calculations	_8-9_________
Recruitment	15	Strategies for achieving adequate participant enrolment to reach target sample size	_8___________

Methods: Assignment of interventions (for controlled trials)

Allocation:

Sequence generation	16a	Method of generating the allocation sequence (eg, computer-generated random numbers), and list of any factors for stratification. To reduce predictability of a random sequence, details of any planned restriction (eg, blocking) should be provided in a separate document that is unavailable to those who enrol participants or assign interventions	_9___________
Allocation concealment mechanism	16b	Mechanism of implementing the allocation sequence (eg, central telephone; sequentially numbered, opaque, sealed envelopes), describing any steps to conceal the sequence until interventions are assigned	_9___________
Implementation	16c	Who will generate the allocation sequence, who will enrol participants, and who will assign participants to interventions	_9___________
Blinding (masking)	17a	Who will be blinded after assignment to interventions (eg, trial participants, care providers, outcome assessors, data analysts), and how	_9___________
	17b	If blinded, circumstances under which unblinding is permissible, and procedure for revealing a participant's allocated intervention during the trial	______13-14____

Methods: Data collection, management, and analysis

Data collection methods	18a	Plans for assessment and collection of outcome, baseline, and other trial data, including any related processes to promote data quality (eg, duplicate measurements, training of assessors) and a description of study instruments (eg, questionnaires, laboratory tests) along with their reliability and validity, if known. Reference to where data collection forms can be found, if not in the protocol	_13-15_______
	18b	Plans to promote participant retention and complete follow-up, including list of any outcome data to be collected for participants who discontinue or deviate from intervention protocols	__14___________

Data management	19	Plans for data entry, coding, security, and storage, including any related processes to promote data quality (eg, double data entry; range checks for data values). Reference to where details of data management procedures can be found, if not in the protocol	_19__________
Statistical methods	20a	Statistical methods for analysing primary and secondary outcomes. Reference to where other details of the statistical analysis plan can be found, if not in the protocol	_17__________
	20b	Methods for any additional analyses (eg, subgroup and adjusted analyses)	_17____________
	20c	Definition of analysis population relating to protocol non-adherence (eg, as randomised analysis), and any statistical methods to handle missing data (eg, multiple imputation)	_17__________
**Methods: Monitoring**			
Data monitoring	21a	Composition of data monitoring committee (DMC); summary of its role and reporting structure; statement of whether it is independent from the sponsor and competing interests; and reference to where further details about its charter can be found, if not in the protocol. Alternatively, an explanation of why a DMC is not needed	____19_________
	21b	Description of any interim analyses and stopping guidelines, including who will have access to these interim results and make the final decision to terminate the trial	_____20________
Harms	22	Plans for collecting, assessing, reporting, and managing solicited and spontaneously reported adverse events and other unintended effects of trial interventions or trial conduct	_19-20_________
Auditing	23	Frequency and procedures for auditing trial conduct, if any, and whether the process will be independent from investigators and the sponsor	_____19_____
**Ethics and dissemination**			
Research ethics approval	24	Plans for seeking research ethics committee/institutional review board (REC/IRB) approval	_21__________
Protocol amendments	25	Plans for communicating important protocol modifications (eg, changes to eligibility criteria, outcomes, analyses) to relevant parties (eg, investigators, REC/IRBs, trial participants, trial registries, journals, regulators)	_20________

Consent or assent	26a	Who will obtain informed consent or assent from potential trial participants or authorised surrogates, and how (see Item 32)	_21__________
	26b	Additional consent provisions for collection and use of participant data and biological specimens in ancillary studies, if applicable	____NA________ _
Confidentiality	27	How personal information about potential and enrolled participants will be collected, shared, and maintained in order to protect confidentiality before, during, and after the trial	_19___________
Declaration of interests	28	Financial and other competing interests for principal investigators for the overall trial and each study site	_21___________
Access to data	29	Statement of who will have access to the final trial dataset, and disclosure of contractual agreements that limit such access for investigators	______21_______
Ancillary and post-trial care	30	Provisions, if any, for ancillary and post-trial care, and for compensation to those who suffer harm from trial participation	________20______
Dissemination policy	31a	Plans for investigators and sponsor to communicate trial results to participants, healthcare professionals, the public, and other relevant groups (eg, via publication, reporting in results databases, or other data sharing arrangements), including any publication restrictions	____21_________
	31b	Authorship eligibility guidelines and any intended use of professional writers	_21___________
	31c	Plans, if any, for granting public access to the full protocol, participant-level dataset, and statistical code	__21___________
**Appendices**			
Informed consent materials	32	Model consent form and other related documentation given to participants and authorised surrogates	_Yes_________
Biological specimens	33	Plans for collection, laboratory evaluation, and storage of biological specimens for genetic or molecular analysis in the current trial and for future use in ancillary studies, if applicable	____NA________ _

*It is strongly recommended that this checklist be read in conjunction with the SPIRIT 2013 Explanation & Elaboration for important clarification on the items. Amendments to the protocol should be tracked and dated. The SPIRIT checklist is copyrighted by the SPIRIT Group under the Creative Commons "Attribution-NonCommercial-NoDerivs 3.0 Unported" license.

For peer review only - http://bmjopen.bmj.com/site/about/guidelines.xhtml
